# Long noncoding RNA *CHROMR* regulates antiviral immunity in humans

**DOI:** 10.1073/pnas.2210321119

**Published:** 2022-08-24

**Authors:** Coen van Solingen, Yannick Cyr, Kaitlyn R. Scacalossi, Maren de Vries, Tessa J. Barrett, Annika de Jong, Morgane Gourvest, Tracy Zhang, Daniel Peled, Raadhika Kher, MacIntosh Cornwell, Michael A. Gildea, Emily J. Brown, Stephanie Fanucchi, Musa M. Mhlanga, Jeffrey S. Berger, Meike Dittmann, Kathryn J. Moore

**Affiliations:** ^a^NYU Cardiovascular Research Center, Leon H. Charney Division of Cardiology, Department of Medicine, New York University Langone Health, New York, NY 10016;; ^b^Department of Microbiology, New York University Langone Health, New York, NY 10016;; ^c^Institute for Systems Genetics, Department of Medicine, New York University Langone Health, New York, NY 10016;; ^d^Lemba Therapeutics B.V., 6525 GA Nijmegen, The Netherlands;; ^e^Epigenomics & Single Cell Biophysics Group, Department of Cell Biology FNWI, Radboud University, Nijmegen, 6500 HB, The Netherlands;; ^f^Department of Human Genetics, Radboud University Medical Center, Nijmegen, 6500 HB, The Netherlands;; ^g^Radboud Institute for Molecular Life Sciences, Nijmegen, 6500 HB, The Netherlands;; ^h^Department of Cell Biology, New York University School of Medicine, New York, NY 10016

**Keywords:** lncRNA, innate immune signaling, interferon-stimulated genes, antiviral response

## Abstract

Long noncoding RNAs (lncRNAs) have emerged as critical regulators of gene expression, yet their contribution to immune regulation in humans remains poorly understood. Here, we report that the primate-specific lncRNA *CHROMR* is induced by influenza A virus and SARS-CoV-2 infection and coordinates the expression of interferon-stimulated genes (ISGs) that execute antiviral responses. *CHROMR* depletion in human macrophages reduces histone acetylation at regulatory regions of ISG loci and attenuates ISG expression in response to microbial stimuli. Mechanistically, we show that *CHROMR* sequesters the interferon regulatory factor (IRF)-2-dependent transcriptional corepressor IRF2BP2, thereby licensing IRF-dependent signaling and transcription of the ISG network. Consequently, *CHROMR* expression is essential to restrict viral infection of macrophages. Our findings identify *CHROMR* as a key arbitrator of antiviral innate immune signaling in humans.

## Significance

An effective innate immune response to virus infection requires the induction of type I interferons and up-regulation of hundreds of interferon-stimulated genes (ISGs) that instruct antiviral functions and immune regulation. Deciphering the regulatory mechanisms that direct expression of the ISG network is critical for understanding the fundamental organization of the innate immune response and the development of antiviral therapies. We define a regulatory role for the primate-specific long noncoding RNA *CHROMR* in coordinating ISG transcription. *CHROMR* sequesters the interferon regulatory factor (IRF)-2/IRF2BP2 complex that restrains ISG transcription and thus is required to restrict influenza virus replication. These data identify a novel regulator of the antiviral gene program in humans and provide insights into the multilayered regulatory network that controls the innate immune response.

Human respiratory viruses, including influenza A virus and severe acute respiratory syndrome coronavirus 2 (SARS-CoV-2), are major causes of morbidity and mortality worldwide. Effective antiviral immunity relies on the activation of conserved innate immune signaling pathways that coordinate the production of type I interferons (IFNα/β) and the expression of several hundred interferon-stimulated genes (ISGs), which collectively subvert viral entry, replication, and pathogenesis (1). IFNα/β are secreted cytokines that bind IFNα/β receptors to initiate Janus kinase (JAK)–signal transducers and activators of transcription (STAT) signaling and the assembly of the ISG factor 3 complex, consisting of interferon regulatory factor (IRF)-9 together with a STAT1–STAT2 heterodimer. This complex transcriptionally activates target genes harboring regulatory IFN-stimulated response elements (ISREs), culminating in the expression of hundreds of ISGs (1, 2). In addition, constitutive and IFN-induced ISG expression can be regulated by IRF-1 binding of ISREs (2, 3). Notably, these pathways must be strictly controlled, as dysregulation of IFN production, signaling, or ISG expression can lead to persistent inflammation and autoimmune disorders, such as systemic lupus erythematosus and Aicardi–Goutières syndrome (4).

Emerging evidence suggests that long noncoding RNAs (lncRNAs) critically regulate the expression of protein-coding genes and their interaction networks in diverse biological processes, including innate immunity (5, 6). Defined as RNA transcripts longer than 200 nucleotides that lack protein-coding potential, lncRNAs execute their structural and regulatory functions by interacting with DNA, protein, or other RNAs in the nucleus or cytoplasm. LncRNAs contribute to gene regulation through diverse mechanisms, including through guiding or sequestering chromatin-modifying enzymes and transcriptional complexes in the nucleus; regulating mRNA processing, splicing, and translation; and acting as competitive inhibitors of endogenous RNAs (e.g., microRNAs) or proteins in the cytoplasm (7, 8). To date, a limited number of lncRNAs have been described to regulate the IFN response by altering the function of viral sensors, production of IFNs, and expression of ISGs. For example, *lncATV* (9) and *lncRNA-LSm3b* (10) have been shown to interact with the cytosolic double-stranded RNA (dsRNA) sensor RIG-I and restrict its function, whereas *Lnczc3h7a* promotes RIG-I function by enabling its interaction with TRIM25 (11). Similarly, *lnc-ITPRIP-1* binds and enhances the function of the RIG-I–like receptor MDA5 (12). Other lncRNAs have been shown to be induced by type I IFNs and mediate feedback inhibition of IFN responses, such as *lnc-MxA*, which negatively regulates IFNβ expression by impeding nuclear factor kappa light chain enhancer of activated B cells (NF-κB) and IRF3 binding at its promoter (13), and *LUCAT1*, which binds and sequesters STAT1 in the nucleus to limit IFN signaling (14). *BISPR* is an example of a lncRNA expressed from a bidirectional promoter that cis-regulates expression of its neighboring gene, *BST2* (Tetherin), an ISG that is known to prevent infection (15). *CCR5AS* behaves as a decoy for the RNA-binding protein RALY, preventing its binding to and repression of the chemokine receptor CCR5 (16). Finally, *lncRNA-CMPK2* (17), *NRAV* (18), and *NRIR* (19) have been shown to broadly alter ISG expression, although the exact mechanisms remain unclear.

Most lncRNAs exhibit poor evolutionary conservation, suggesting that functional investigation of human lncRNAs that modulate the IFN response and antiviral immunity may unveil key points of pathogen control and novel targets for therapeutic intervention. In this study we show that *CHROMR*, a primate-specific lncRNA first identified to regulate cellular lipid metabolism (20), is highly induced in the patients infected with the influenza virus or SARS-CoV-2, and in human primary macrophages and cell lines exposed to RNA viruses or the synthetic dsRNA polyinosinic:cytidylic acid (poly[I:C]). Loss-of-function studies identify a critical role for *CHROMR* in the regulation of ISG expression and restriction of influenza A virus replication in macrophages. Although activation of NF-κB signaling is intact in *CHROMR*-depleted macrophages, these cells exhibit reduced expression of an IRF-inducible ISRE luciferase reporter gene, indicating a defect in transcriptional activation of IRF signaling and interferon response pathways. In mechanistic studies, we identify that *CHROMR* sequesters the nuclear transcriptional corepressor IRF2BP2, which acts together with IRF-2 to repress ISG transcription, thereby licensing IRF-dependent signaling and transcription of the ISG network. Collectively, our data provide insights into the multilayered regulatory network that controls ISG expression and the innate immune response to viruses.

## Results and Discussion

### LncRNA *CHROMR* Associates with the Interferon Response in Patients with COVID-19 and Influenza.

**Fig. 1.**
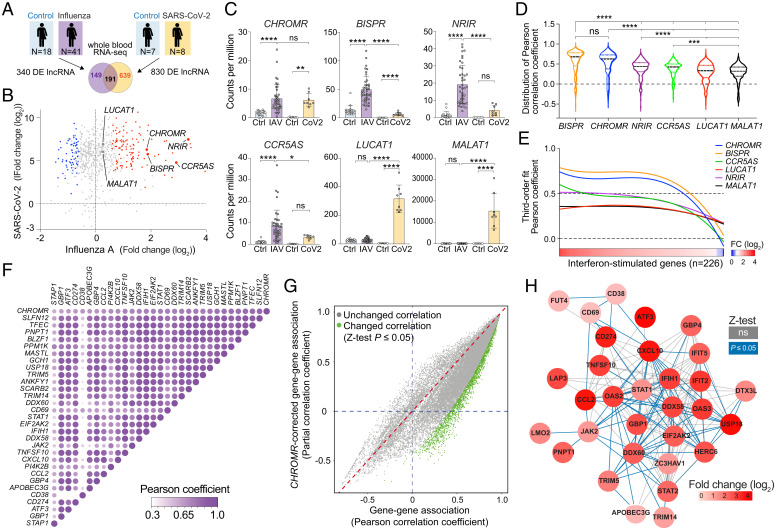
LncRNA *CHROMR* is up-regulated in patients infected with SARS-CoV-2 or IAV and correlates with transcriptional activation of antiviral gene programs. (*A*) Experimental design for identification of lncRNAs differentially expressed in whole blood of patients with IAV or SARS-CoV-2 and controls. (*B*) Scatter plot of the lncRNAs identified as commonly dysregulated in patients infected with SARS-CoV-2 and IAV by whole blood high-throughput RNA-seq. Up-regulated lncRNAs are indicated in red (*n* = 116) and down-regulated in blue (*n* = 75); −1.5 < fold change > 1.5; *P*-adj < 0.05. Nonsignificantly changed lncRNAs are indicated in gray. (*C*) Normalized transcript expression (counts per million) of *CHROMR* and lncRNAs described to regulate interferon responses in blood of patients infected with IAV (*n* = 41) or SARS-CoV-2 (CoV2, *n* = 8) and of control subjects (*n* = 18, *n* = 7, respectively). (*D*) Violin plot showing the distributions of the Pearson correlation coefficient between indicated lncRNAs and 226 differentially expressed ISGs common to IAV- and SARS-CoV-2–infected patients. (*E*) Robust third-order nonlinear fit of the lncRNA × ISG Pearson correlation coefficient displayed as a function of the differential expression of the ISGs. (*F*) Pearson correlation matrix showing the 30 ISGs that are most strongly associated with *CHROMR* in whole blood in IAV-infected patients. (*G*) Scatter plot of the Pearson coefficients of bivariate correlations between the 226 ISGs and the corresponding *CHROMR*-corrected partial correlation coefficients, with correlations that are significantly changed by correcting for *CHROMR* expression highlighted in green. . (*H*) *CHROMR*-associated ISG interactome clustered on the basis of functional relationship (edges); blue-colored lines represent functional correlations that were significantly changed upon *CHROMR* correction by partial correlation analysis, and gray-colored lines represent nonsignificantly changed bivariate correlations. Red shading corresponds to fold change observed in whole blood RNA-seq as shown in (*B*). Data are mean ± SEM (*C*) or ± quartiles (*D*), third-order polynomial nonlinear fit with robust adjustment (*E*). *P* values were calculated via one-way ANOVA, with Sidak’s multiple comparison test (*C*) or Kruskal–Wallis test with Dunn’s correction for multiple comparison (*D*). All bivariate and partial correlation analyses performed in IAV-infected patients (*n* = 41) (*D*–*H*). All data log_1p_ transformed for linear regression analysis (*F*–*H*). Difference in correlation coefficient assessed by Fisher *r*-to-*Z* transformation followed by *Z*-test (*G*). *P ≤ 0.05; **P < 0.01; ***P < 0.001; ****P < 0.0001.

To identify lncRNAs implicated in the host response to respiratory viruses, we performed RNA sequencing (RNA-seq) of whole blood from hospitalized patients with coronavirus disease 2019 (COVID-19) induced by SARS-CoV-2 (*n* = 8) and age- and sex-matched controls (*n* = 7) (*SI Appendix*, Table S1), and compared this to whole blood transcriptomic analysis of subjects with influenza (IAV; *n* = 41) and controls (*n* = 18) (retrieved from GSE157240 (21)). Differential expression analysis revealed 830 lncRNAs altered in patients with COVID-19 and 340 changed in patients with influenza; 191 lncRNAs were dysregulated in both diseases (*P*-adj < 0.05, −1.5 < fold change > 1.5; Fig. 1 *A* and *B* and *SI Appendix*, Table S2). Among the top mutually up-regulated lncRNAs, we identified *CHROMR* (alias *CHROME*) (Fig. 1 *B* and *C*), a primate-specific lncRNA previously identified to regulate cellular lipid metabolism (20). Of note, levels of *CHROMR* strongly correlated with ISGs differentially expressed (*n* = 226) in COVID-19 and influenza patients compared to controls (Fig. 1*D*), in addition to previously associated lipid metabolism genes (*SI Appendix*, Fig. S1*A*). When compared to other human lncRNAs known to regulate antiviral responses (14–16, 19, 22), *CHROMR* showed a distribution of correlation coefficients equivalent to *BISPR* (lncBST2) and significantly higher than *NRIR*, *CCR5AS*, *LUCAT1*, and *MALAT1* (Fig. 1*D*). Of these lncRNAs, *CHROMR* showed an equivalent transcriptional response to both viral infections (Fig. 1*C*). To further visualize the association of *CHROMR* with ISGs differentially expressed after influenza A virus or SARS-CoV-2 infection, we rank-ordered the ISGs by level of differential expression in influenza A virus –infected patients and plotted their correlation coefficient with *CHROMR.* Using a robust third-order nonlinear regression analysis, we observed that *CHROMR* associates strongly with genes that are up-regulated by ≥2-fold change (mean *r ≥* 0.5), similar to that observed with *BISPR*, whereas *CCR5AS*, *NRIR*, *LUCAT1*, and *MALAT1* did not exhibit a distinct pattern of association with the continuum of differentially expressed ISGs (Fig. 1*E*). Among the top 30 ISGs most correlated with *CHROMR*, we observed that 453 of 465 ISG × ISG pairs are significantly associated (Fig. 1*F*). To investigate whether *CHROMR* influences ISG × ISG associations as a covariate, we compared the bivariate correlation coefficient of all 226 differentially expressed ISGs to their corresponding *CHROMR*-corrected partial correlation coefficient. The majority of the 25,425 ISG × ISG correlation coefficients generated were decreased by *CHROMR* correction, with 1,845 significantly decreased (Fisher’s *r*-to-*Z* transformation followed by *Z*-test; Fig. 1*G*). Of note, only one ISG × ISG correlation coefficient was significantly increased, indicating that *CHROMR* has a robust positive impact on the correlation between ISGs. We next generated a functional interactome of the 50 genes whose expression is most associated with *CHROMR* (*SI Appendix*, Fig. S1*B*). Of these, 31 genes were functionally related, representing a total of 172 ISG × ISG associations (all edges, Fig. 1*H*), 79 of which were statistically altered upon correction for *CHROMR* (blue-colored edges, Fig. 1*H*). Taken together, these results indicate a role for *CHROMR* in directing the coordinated ISG response to SARS-CoV-2 and influenza A infection.

**Fig. 2.**
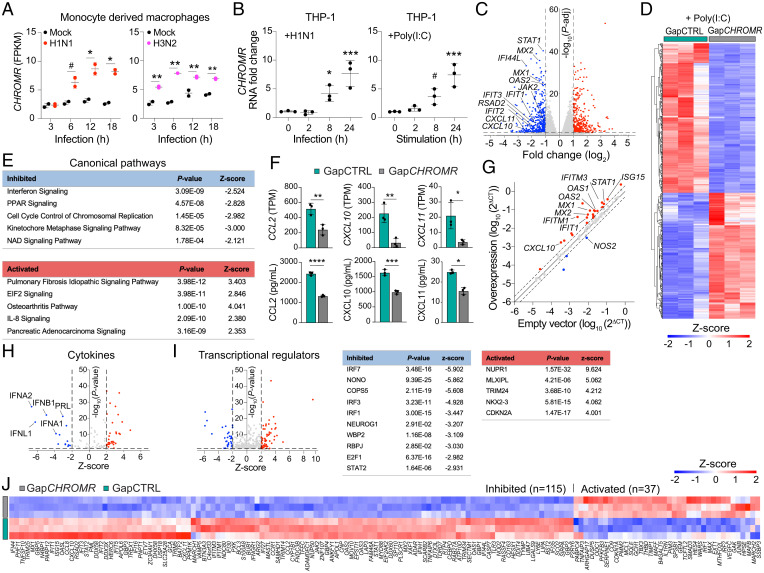
*CHROMR* deficiency leads to diminished expression of ISGs. (*A*) Time course of *CHROMR* expression (FPKM) in human monocyte-derived macrophages infected with A/California/04/09 (H1N1) virus, A/Wyoming/03/03 (H3N2) virus, or mock infected. (*B*) qPCR analysis of *CHROMR* in human THP-1 macrophages infected with A/WSN/1933 (H1N1) virus(1,000 PFU) or stimulated with synthetic dsRNA poly(I:C) (1 μg/mL). (*C*) Volcano plot showing differentially expressed genes in *CHROMR*-depleted (Gap*CHROMR*-treated) and control (GapCTRL-treated) THP-1 macrophages after poly(I:C) (1 μg/mL, 8 h) stimulation and RNA-seq. Dashed lines indicate fold change (log_2_) = ±1; *P*-adj = 0.05; red dots indicate up-regulated genes; blue dots indicate down-regulated genes; gray dots indicate nonsignificantly changed genes. (*D*) Hierarchical clustering heatmap showing normalized gene expression values in THP-1 macrophages treated with Gap*CHROMR* or GapCTRL in poly(I:C) stimulated conditions (1 μg/mL, 8h). Cutoffs used for visualization: −2 < fold change > 2; and *P*-adj < 0.05. (*E*) List of most affected canonical pathways identified through Ingenuity Pathway Analysis of (*C*) ranked by *P*-adj. (*F*) Expression of top chemokine genes differentially regulated in *CHROMR*-depleted and control THP-1 macrophages. *Top row*: RNA-seq normalized expression counts (TPM) after poly(I:C) (1 μg/mL, 8h). *Bottom row*: Immunoassay of protein levels after poly(I:C) (1 μg/mL, 24h). (*G*) Gene expression profiling of 84 interferon-stimulated genes in THP-1 macrophages stably overexpressing *CHROMR* or an empty vector control. Up-regulated genes are indicated in red and down-regulated genes in blue. Genes indicated are *P* < 0.1. (*H* and *I*) Predicted cytokine (*H*) and transcriptional regulators (*I*) of differentially expressed genes in (*C*); dashed lines indicate *Z*-score = ±2 and *P*-adj = 0.05; red and blue dots indicate significantly up-regulated and down-regulated factors, respectively. (*J*) Hierarchical clustering heatmap showing *Z*-scores of differentially expressed ISGs in *CHROMR*-depleted and control THP-1 macrophages treated with poly(I:C) (1 μg/mL) for 8h, *P*-adj < 0.05. Data are mean ± SEM for 2 (*A*), 3 (*B*–*F* [*Top*], *G*–*J*) independent experiments, or representative of 3 independent experiments (*F* [*Bottom*]). *P* values were calculated via repeated measures two-way ANOVA with Sidak’s multiple comparison test (*A*), one-way ANOVA with Dunnett’s multiple comparison test (*B*), right-tailed Fisher’s exact test (*E*, *H*, and *I*), or two-tailed unpaired Student’s *t* test (*F* and *G*). ^#^*P* < 0.1; *P < 0.05; **P < 0.01; ***P < 0.001; ****P < 0.0001.

To understand how *CHROMR* is regulated in myeloid cells during viral infection, we interrogated RNA-seq data from human monocyte-derived macrophages infected with A/California/04/09 (H1N1), influenza A/Wyoming/03/03 (H3N2), or influenza A/Vietnam/1203/2004 (H5N1) HaLo viruses (retrieved from GSE97672 (23)). We observed that *CHROMR* expression was higher within 3–6 h after IAV infection compared with mock treatment (Fig. 2*A* and *SI Appendix*, Fig. S2*A*). Similar findings were observed in human THP-1 monocyte-derived macrophages infected with A/WSN/1933 (H1N1) virus or treated with the viral mimic poly(I:C), a synthetic dsRNA that activates TLR3 (Fig. 2*B*). To assess the impact of *CHROMR* depletion on the transcriptional response to innate immune stimulation, we knocked down *CHROMR* in THP-1 macrophages by using GapmeR antisense oligonucleotides (Gap*CHROMR*) or control GapmeRs (GapCTRL) and treated with poly(I:C) for 8 h. We confirmed that *CHROMR* expression was diminished upon treatment with Gap*CHROMR* when compared to GapCTRL by quantitative real-time PCR (qPCR) (*SI Appendix*, Fig. S2*B*). Transcriptome profiling by RNA-seq and unsupervised hierarchical clustering of genes differentially expressed in *CHROMR*-sufficient and -depleted macrophages revealed that *CHROMR* silencing markedly reprogrammed transcriptional responses to poly(I:C), with 488 genes showing lower transcript levels and 395 genes showing higher transcript levels compared to GapCTRL treated cells (−2 < fold change > 2, *P*-adj < 0.05; Fig. 2 *C* and *D*). Ingenuity Pathway Analysis of genes differentially expressed upon *CHROMR* knockdown identified “Interferon signaling” as the most repressed canonical pathway, followed by “PPAR signaling” and “Cell cycle control of replication” (Fig. 2*E*). The most differentially regulated genes included the antiviral response genes *IFIT1*, *IFIT2*, *IFIT3*, *RSAD2*, *MX1*, *MX2*, *IFI44L*, and *STAT1* (Fig. 2*C* and *SI Appendix*, Table S3). In addition, we noted that transcript levels of several IFN-induced chemokines were reduced in *CHROMR*-depleted macrophages treated with poly(I:C), including members of the CXCL (*CXCL10*, *CXCL11*) and CCL (*CCL2*) families, a finding that was further validated at the protein level by bead-based immunoassay (Fig. 2*F*). A similar down-regulation of ISGs was observed in *CHROMR*-depleted macrophages treated with bacterial lipopolysaccharide (LPS) (*SI Appendix*, Fig. S2 *D*–*J* and *SI Appendix*, Table S4). Conversely, overexpression of *CHROMR* in THP-1 macrophages increased expression of ISGs, including *CXCL10*, *IFIT1*, *IFITM1*, *IFITM3*, *MX1*, *MX2*, *OAS1*, *OAS2*, *STAT1*, and *ISG15* (Fig. 2*G* and *SI Appendix*, Fig. S2*C* and *SI Appendix*, Table S5), as assessed via a qPCR array to profile 84 selected ISGs.

**Fig. 3.**
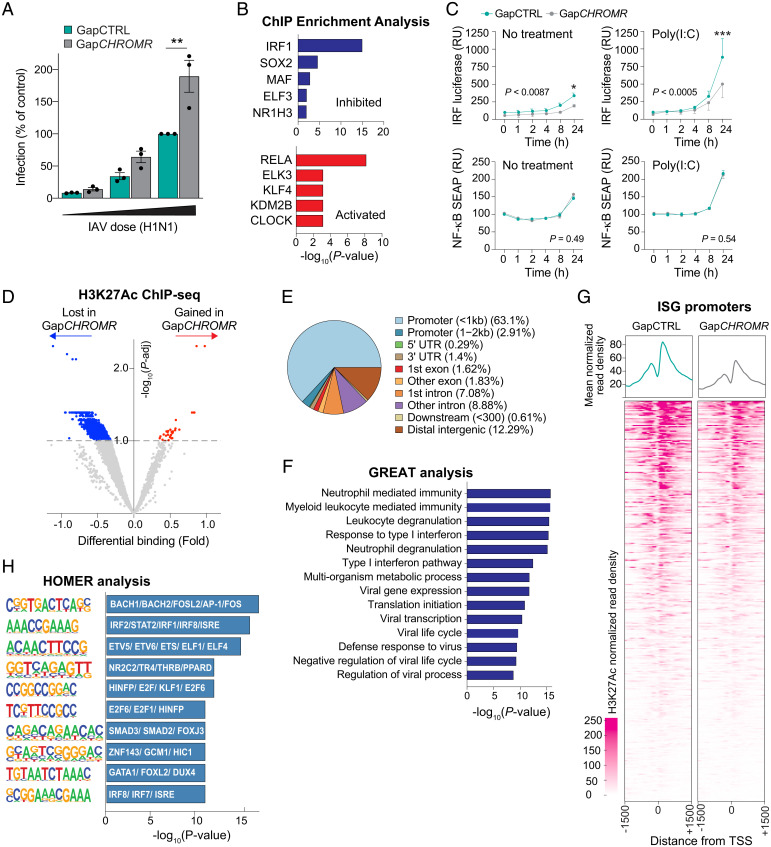
*CHROMR* is required to restrict influenza virus and activate ISG transcription. (*A*) Percentage of viral infection in *CHROMR*-depleted (Gap*CHROMR*-treated) and control (GapCTRL-treated) THP-1 macrophages challenged with A/WSN/1933 (H1N1) virus at increasing doses (100, 500 or 1,000 PFU). Percentages were calculated relative to GapCTRL transfection at highest infection rate. (*B*) Transcription factor binding enrichment scores for ISGs differentially expressed in *CHROMR*-depleted and control THP-1 macrophages stimulated with poly(I:C) via ChIP enrichment analysis (ChEA 2016) database gene set library. (*C*) Reporter assay for IRF-driven transcription (*Top*: luciferase; RU, relative units) or NF-κB–driven transcription (*Bottom*: SEAP, secreted alkaline phosphatase) in THP-1 Dual Reporter macrophages transfected with Gap*CHROMR* or GapCTRL and left untreated or stimulated with poly(I:C) (1 μg/mL). Relative expression is normalized to time 0 (=100). (*D*) Volcano plot showing differential H3K27Ac modification in *CHROMR*-depleted and control THP-1 macrophages stimulated with poly(I:C). ChIP-seq reads that are gained or lost after *CHROMR* knockdown are indicated in red and blue, respectively. Dashed line indicates *P*-adj < 0.1. (*E*) Genomic distribution of H3K27Ac marks lost after CHROMR knockdown identified in *D*, *P*-adj < 0.1. UTR, untranslated region. (*F*) List of biological processes identified via Genomic Regions Enrichment Annotations Tool (GREAT) analysis of H3K27Ac-depleted promoter regions. (*G*) Metagene plots showing the mean (*Top*) and individual unique positions (*Bottom*) of normalized H3K27Ac read density around the transcription start site (TSS ± 1,500 base pairs) of ISGs in THP-1 macrophages transfected with Gap*CHROMR* or GapCTRL. (*H*) Hypergeometric Optimization of Motif EnRichment (HOMER) analysis of promoter regions depleted of H3K27Ac after *CHROMR* knockdown, showing transcription factors with highest similarity score in motif indicated in bars. Data are mean ± SEM for three independent experiments. *P* values were calculated via repeated measures two-way ANOVA with Sidak’s multiple comparison test (*A* and *C*) or binomial test (*B*, *F*, and *H*). *P ≤ 0.05; **P < 0.01; ***P < 0.001.

To identify potential factors driving *CHROMR*-associated transcriptional changes, we used Ingenuity Pathway Analysis to ascertain upstream regulators of genes differentially expressed upon *CHROMR* knockdown, including cytokines and transcription factors shown experimentally to alter the affected gene pathways. This analysis predicted repression of interferons, including type I (IFNα, IFNβ) and type III (IFNλ), in *CHROMR*-depleted THP-1 macrophages stimulated with poly(I:C) and LPS (Fig. 2*H* and *SI Appendix*, Fig. S2*I*). Accordingly, transcription factors associated with interferon signaling were predicted to be inhibited upon *CHROMR* knockdown, including IRF-1, IRF-3, and IRF-7, and STAT proteins, which transduce signaling from the IFN receptors (Fig. 2*I* and *SI Appendix*, Fig. S2*J*). Consistent with the established roles of IRF-1 and JAK-STAT signaling in the transcriptional regulation of ISGs, we noted that 115 of 389 ISGs induced by poly(I:C) and 230 of 389 ISGs induced by LPS were expressed at lower levels in Gap*CHROMR*-treated compared to GapCTRL-treated THP-1 macrophages (Fig. 2*J* and *SI Appendix*, Fig. S3*A*). Prominent among these ISGs down-regulated upon *CHROMR* knockdown were genes important for pathogen sensing (*TLR3*, *DDX58*/RIG-I, *IFIH1*/MDA5, *IFI16*), as well as inhibition of viral entry (*MX1*, *IFITM1*, *IFITM2*, *TRIM5*), replication (*IFIT*, *ISG15*, *OAS1*), and budding (*RSAD2*/Viperin, *BST2*/Tetherin). As these data suggest *CHROMR* is a critical component of the antiviral response, we next assessed the impact of *CHROMR* knockdown on influenza A virus replication. THP-1 macrophages were treated with Gap*CHROMR* or GapCTRL and challenged with influenza A/WSN/1933 (H1N1) virus at increasing dosages of 100, 500 or 1,000 plaque forming units (PFUs) per well for multicycle replication. *CHROMR* knockdown significantly increased IAV infection levels in Gap*CHROMR*-treated compared to GapCTRL-treated macrophages, suggesting a pivotal role for *CHROMR* in restricting IAV infection (Fig. 3*A* and *SI Appendix*, Fig. S3*D*).

### *CHROMR* Associates with Chromatin and Shapes H3K27Ac at ISG Regulatory Regions.

As *CHROMR* is known to regulate lipid metabolism by sequestering microRNAs in the cytoplasm (20), we performed *in silico* analyses to predict microRNA regulators of the ISGs differentially expressed upon *CHROMR* knockdown (*SI Appendix*, Fig. S3*B*). miR-21 and miR-184 were identified as putative repressors of genes whose expression was reduced in Gap*CHROMR*-treated compared to GapCTRL-treated macrophages; however, *CHROMR* lacks binding sites for these microRNAs, suggesting an alternative mechanism of gene regulation. Cell fractionation studies revealed that *CHROMR* localizes to the nucleus as well as the cytoplasm of macrophages (*SI Appendix*, Fig. S4*A*). Thus, we assessed whether *CHROMR* associates with chromatin by performing RNA immunoprecipitation of histone H3. qPCR of H3 immunoprecipitates showed enrichment of *CHROMR*, particularly variants 1, 3 and 4, at levels similar to another chromatin-binding lncRNA, *NEAT1* (24) (*SI Appendix*, Fig. S4*B*). As many nuclear lncRNAs can act *in cis* to regulate adjacent loci, we considered whether *CHROMR* could regulate genes within its topologically associating domain (TAD) (*SI Appendix*, Fig. S4*C*). Chromatin Interaction Analysis by Paired-End Tag Sequencing, which combines chromatin immunoprecipitation (ChIP)-based methods, chromatin proximity interaction, and chromosome conformation capture, revealed weak interactions between *CHROMR* and its neighboring genes, including the *PRKRA* gene that encodes protein activator of the interferon-induced protein kinase PKR, which binds dsRNA and activates RIG-I–mediated antiviral signaling (*SI Appendix*, Fig. S4 *D* and *E*). However, the frequency of those interactions was only marginally higher than that observed with genes in distant TADs, suggesting that *CHROMR*’s TAD is not highly insulated. Furthermore, we observed no difference in the expression of genes within *CHROMR*’s TAD, including *PRKRA*, in Gap*CHROMR* and GapCTRL treated THP-1 macrophages, discounting gene regulation *in cis* as the mechanism of *CHROMR*’s effect on interferon signaling (*SI Appendix*, Fig. S4*F*).

To further investigate the role of *CHROMR* in transcriptional activation of ISGs, we performed gene set enrichment analysis of ISGs differentially expressed in Gap*CHROMR-*treated and GapCTRL-treated macrophages stimulated with poly(I:C) or LPS by using the chromatin immunoprecipitation-X enrichment analysis gene set library (25). ISGs down-regulated upon *CHROMR* depletion and subsequent microbial stimulus were most significantly enriched in ChIP experiments for IRF-1 (Fig. 3*B* and *SI Appendix*, Fig. S3*C*), a transcription factor that regulates constitutive expression of antiviral genes and induction of the early antiviral response (26). Our analysis also identified the nuclear hormone receptor NR1H3/LXRA, which controls transcription of lipid homeostasis genes previously linked to *CHROMR* (27). To test whether *CHROMR* regulates IRF activation, we used THP-1 macrophages stably expressing IRF- and NF-κB–inducible reporter genes. While we observed no difference in NF-κB reporter activation in Gap*CHROMR* or GapCTRL-treated macrophages, *CHROMR* knockdown reduced IRF reporter activity in both unstimulated and poly(I:C)-stimulated THP-1 macrophages (Fig. 3*C*). IRF1 mediates both constitutive and inducible expression of host defense genes and is particularly important for regulation of *IFI27*, *OAS2*, *OASL*, and *IFI44* in the basal state (28, 29). Consistent with this, we observed reduced transcript levels of *IFI44*, *OAS2*, and *STAT1* in unstimulated THP-1 macrophages after *CHROMR* knockdown (*SI Appendix*, Fig. S4*G*). Since these studies suggested a defect in constitutive and induced IRF activation in the absence of *CHROMR*, we next assessed the genome-wide distribution of histone H3 lysine 27 acetylation (H3K27Ac), an epigenetic mark of transcriptional activity, in THP-1 macrophages treated with Gap*CHROMR* or GapCTRL. ChIP sequencing (ChIP-seq) showed that *CHROMR* knockdown resulted in depletion of H3K27Ac at 2,753 genomic sites and enrichment of H3K27Ac at 30 sites compared to control macrophages (Fig. 3*D*). Classification of the H3K27Ac peak distribution among genomic features showed that the depletion of H3K27Ac marks after *CHROMR* knockdown occurred mainly in promoter regions (66%), followed by distal intergenic and intronic regions (Fig. 3*E*). Genomic Regions Enrichment of Annotations Tool analysis of genes exhibiting decreased H3K27Ac revealed enrichment of biological processes related to antiviral immunity, including “Response to type I interferon”, “Defense response to virus”, and “Negative regulation of viral life cycle” (Fig. 3*F*). Indeed, characterization of the H3K27Ac read distribution across the transcriptional start site (±1,500 bp) of all ISGs showed markedly reduced H3K27Ac read density in Gap*CHROMR*-treated versus GapCTRL-treated macrophages (Fig. 3*G*), consistent with lower transcription of ISGs in the absence of *CHROMR*. H3K27Ac helps shape active promoters and enhancers by opening chromatin to allow binding of transcriptional regulators. Notably, Hypergeometric Optimization of Motif EnRichment analysis of transcription factor binding motifs within regions of decreased H3K27Ac after *CHROMR* knockdown identified an ISRE motif predicted to bind IRF-1 and its functional antagonist IRF-2 (Fig. 3*H*), suggesting that *CHROMR* may shape active ISG promoters by facilitating IRF-1 recruitment.

### *CHROMR* Binds IRF-2 Binding Protein 2.

**Fig. 4.**
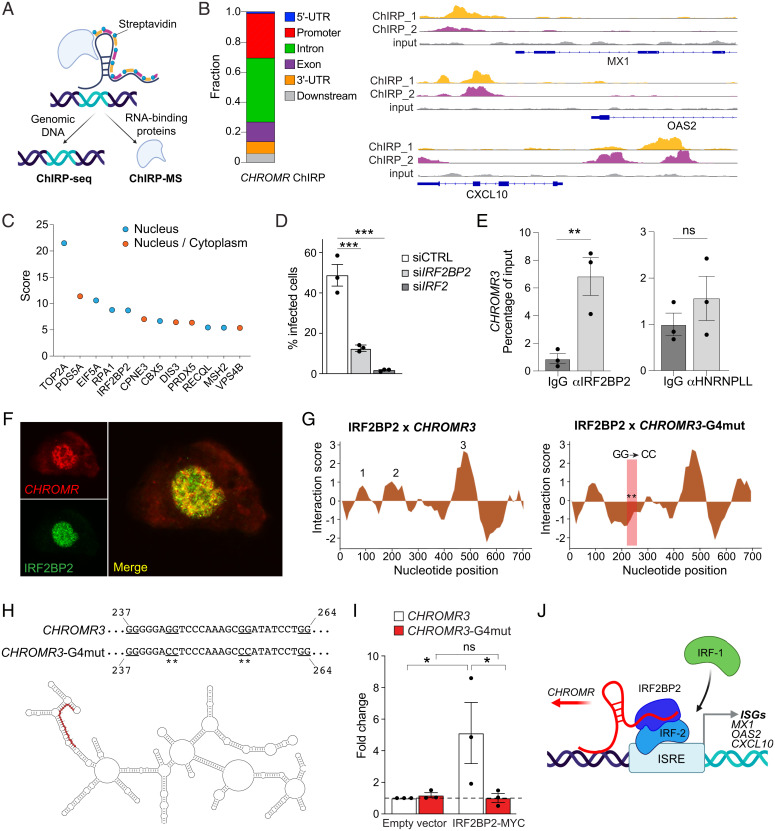
*CHROMR* binds to IRF2BP2 to control interferon-stimulated gene expression. (*A*) Schematic representation of ChIRP followed by genomic DNA sequencing (ChIRP-Seq) or mass spectrometry (ChIRP-MS) to identify RNA-binding proteins. (*B*) Distribution of *CHROMR* binding sites within ISG loci (*Left*) and representative ChIRP-seq reads (*Top*: “even” probe set [ChIRP_1]; *Middle*: “odd” probe set [ChIRP_2]; *Bottom*: input) at selected ISG promoters (*Right*). UTR, untranslated region. (*C*) Nuclear *CHROMR*-binding proteins identified by ChIRP-MS in THP-1 macrophages from three independent experiments. (*D*) Percentage of cells infected with IAV/WSN/1933 (H1N1, 1,000 PFU) in THP-1 macrophages transfected with siRNAs against *IRF2BP2*, *IRF2*, or a nontargeting siRNA control (siCTRL). (*E*) qPCR analysis of *CHROMR3* in RNA immunocomplexes precipitated from THP-1 macrophages with IRF2BP2 or HNRNPLL antibodies or IgG as a control. (*F*) Representative microscopic image of RNA fluorescence in situ hybridization staining for *CHROMR* (red) in combination with immunofluorescent staining for IRF2BP2 in THP-1 macrophages. Merged image indicates signal colocalization (yellow). (*G*) catRAPID predicted interaction profile of IRF2BP2 with *CHROMR3* or *CHROMR3*-G4 mutant (position of mutation indicated by ** in boxed region). (*H*) Visualization of *CHROMR3* secondary structure in RNArtist, with the putative IRF2BP2–G-quadruplex interaction domain highlighted in red (*Bottom*). Site-directed mutation of the putative G-quadruplex (underlined) in *CHROMR3* (*Top*). (*I*) Relative enrichment of *CHROMR3* or *CHROMR3*-G4mut in MYC-IRF2BP2 immunoprecipitates. (*J*) Integrated model depicting *CHROMR* binding to IRF2BP2 to sequester the IRF-2 repressor complex from ISREs, facilitating access for activating interferon regulatory factors (e.g., IRF-1). (*E* and *I*) Data are relative to IgG control; mean ± SE of three independent experiments. *P* values were calculated via one-way ANOVA with Dunnett’s multiple comparison test (*D* and *I*) or a repeated-measures two-way ANOVA with Sidak’s multiple comparison test (*E*). *P ≤ 0.05; **P < 0.01; ***P < 0.001.

To examine the possibility that *CHROMR* acts *in trans* to regulate expression of ISGs, we performed chromatin isolation by RNA purification (ChIRP) of endogenous *CHROMR* in nuclear extracts of crosslinked THP-1 macrophages by using two independent pools of biotinylated *CHROMR*-specific antisense RNA probes (Fig. 4*A*). Isolation of *CHROMR*-associated chromatin followed by DNA sequencing (ChIRP-seq) revealed enrichment of *CHROMR* at 237 of 389 known ISGs, most prominently within promoter and intronic regions (Fig. 4*B*). As examples, genomic regions near the transcription start site of *CXCL10*, *OAS2*, and *MX1*, genes that were particularly affected by *CHROMR* gain or loss of function (Fig. 2 *C* and *G*), showed binding of both pools of *CHROMR*-specific probes (Fig. 4*B*). These findings suggest that *CHROMR* binds, either directly or indirectly, to regulatory regions of ISGs to promote their transcription. To better understand how *CHROMR* might mediate this effect, we performed comprehensive identification of RNA-binding proteins by mass spectrometry (ChIRP-MS) (30) in THP-1 macrophages to identify *CHROMR*-interacting proteins. We identified 26 proteins that coprecipitated with *CHROMR*, including 7 nuclear proteins, 14 cytoplasmic proteins, and 5 proteins that localized to both the nucleus and cytoplasm (Fig. 4*C* and *SI Appendix*, Fig. S5*A*). Among the nuclear proteins associated with *CHROMR* were two factors previously implicated in the regulation of interferon responses: topoisomerase 2a (31) and interferon regulatory factor-2 binding protein 2 (IRF2BP2) (32, 33). IRF2BP2 is a binding partner of the IRF-2 transcriptional repressor that antagonizes IRF-1–mediated transcriptional activation (32, 34). Thus, we postulated that *CHROMR* may regulate transcriptional activation of ISGs and antiviral responses by sequestering IRF2BP2. Consistent with this possibility, small interfering RNA (siRNA)-mediated knockdown of endogenous IRF2BP2 or IRF-2 inhibited infection with influenza A/WSN/1933 (H1N1) compared to control siRNA treatment in THP-1 macrophages (Fig. 4*D*). The *CHROMR* and IRF2BP2 interaction was confirmed by RNA immunoprecipitation, which showed that *CHROMR* was enriched in IRF2BP2 immunoprecipitates compared to immunoglobulin G (IgG) controls (Fig. 4*E*). To further substantiate this interaction, we combined RNA fluorescence *in situ* hybridization for *CHROMR* with immunofluorescence for IRF2BP2 in THP-1 macrophages and observed nuclear colocalization (Fig. 4*F*). Using the catRAPID algorithm (35), we calculated the binding propensity of *CHROMR* and IRF2BP2, and we predicted protein-binding regions within *CHROMR*. This analysis identified nucleotides 90–141 (domain 1), 177–269 (domain 2), and 468–552 (domain 3) of *CHROMR* as potential IRF2BP2 interaction domains (Fig. 4*G*). Notably, domain 2 of *CHROMR* contains tandem G-rich sequences predicted by QGRSmapper (36) to form a G-quadruplex (G4) secondary structure (*SI Appendix*, Fig. S5*B*), which can enable RNA–protein interactions (37). *In silico* mutation of this putative G4, simulated by replacing two of the four tandem GG doublets with CC doublets, reversed the binding propensity of IRF2BP2 to domain 2 of *CHROMR* (Fig. 4*G*, *Right* panel). To directly test whether *CHROMR* interacts with IRF2BP2 through this putative G4, we performed site-directed mutagenesis of two G-doublets into CC-doublets in *CHROMR3* (Fig. 4*H*), and overexpressed wild-type (WT) or G4-mutant *CHROMR* with a MYC/DDK-tagged IRF2BP2 in HEK293T cells. We immunoprecipitated IRF2BP2 by using an antibody against MYC/DDK and assessed the presence of *CHROMR* in these complexes by qPCR. Although WT and G4-mutant *CHROMR* were expressed at similar levels (*SI Appendix*, Fig. S5*C*), only WT *CHROMR* was enriched in IRF2BP2 immunoprecipitates (Fig. 4*I*), indicating that the G-rich sequence in domain 2 is required for *CHROMR*-IRF2BP2 binding.

Taken together, our work unveils a critical role for *CHROMR* in coordinating ISG expression and antiviral immunity in humans. By combining human transcriptomic profiling in influenza A and SARS-CoV-2 infection with *in vitro* mechanistic studies, we demonstrated that *CHROMR* regulates the antiviral gene program by sequestering the nuclear IRF-2/IRF2BP2 repressor complex, thereby releasing its inhibitory effect on transcription of ISGs (Fig. 4*J*). These findings expand *CHROMR*’s previously identified role as a competing endogenous RNA that regulates cholesterol efflux and fatty acid oxidation via microRNA sequestration in the cytoplasm. Many viruses rewire host lipid synthesis and metabolism to facilitate replication (38, 39), and thus, increased *CHROMR* expression in virus-infected cells would both mitigate cellular lipid accumulation and increase ISG transcription to mount an antiviral immune response. As *CHROMR* is not conserved in common preclinical animal models used to study antiviral immunity, our findings underscore the merit of investigating primate-specific lncRNAs to decipher novel regulatory mechanisms that govern host defense and immunopathology in human viral infections, such as influenza and COVID-19. Further studies of *CHROMR* expression and function in human pathological states in which the interferon signaling response is diminished (e.g., severe COVID-19) or elevated (e.g., systemic lupus erythematosus) may reveal possibilities for therapeutic targeting.

## Materials and Methods

The detailed methods for cell culture, RNA isolation, cell fractionation and qPCR, RNA-seq, ChIP-seq, ChIRP-seq, ChIRP-MS, gene expression profiling, quantification of IAV infection, dual reporter assays, RNA immunoprecipitation, RNA fluorescence *in situ* hybridization, mutagenesis studies, and bioinformatics can be found in *SI Appendix*, *SI Materials and Methods*.

### Human Studies.

A cohort of eight hospitalized patients with COVID-19 were recruited from New York University (NYU) Langone Health between May 11 and 21, 2020. SARS-CoV-2 infection was confirmed by qPCR, in accordance with current standards. All patients with COVID-19 and age- and sex-matched control donors were recruited under study protocols approved by the NYU Langone Health Institutional Review Board. No exclusion criteria were applied. Each study participant or their legal authorized representative gave written informed consent for study enrollment in accordance with the Declaration of Helsinki. For patients with COVID-19, enrollment criteria included age greater than 18, hospital admission, positive SARS-CoV-2 testing, and informed consent. Patients with COVID-19 were monitored until discharge or death. Demographics of the cohort are listed in *SI Appendix*, Table S1.

### Whole Blood Transcriptome Profiling.

Whole blood of patients with COVID-19 (*n* = 8) and controls (*n* = 7) was collected into PAXgene Blood RNA tubes (PreAnalytiX GmbH, BD Biosciences), and RNA was isolated. The quality and yield of the isolated RNA were determined with an Agilent 2100 Bioanalyzer (Agilent) before RNA-seq. RNA library preps were made (Low Input Clontech SMART-seq) and sequenced in single-end mode at the Genome Technology Center at NYU Langone Health with an Illumina NovaSeq 6000.

### Transcriptomic Analysis.

To obtain differentially expressed transcripts between influenza A–infected patients (*n* = 41) and controls (*n* = 18) we queried publicly available dataset GSE157240 (21). FASTQ files from RNA-seq (influenza A and SARS-CoV-2) were processed via the Seq-*N*-Slide pipeline (40). Reads were aligned to the hg38 genome in STAR (41) v2.6.1 and quantified with featureCounts (42) v1.6.3. Read quality was assessed in FASTQC (43) v0.11.7. All downstream analysis was performed in R (44) v3.6.1. Differential expression analysis was performed via DESeq2 (45) v1.24. RNA-seq data derived from whole blood RNA-seq in patients with SARS-CoV-2 are deposited in the Gene Expression Omnibus (accession no. GSE190413).

Differentially expressed lncRNA within both datasets were identified with the lncRNA biotype-annotation within the Ensembl gene annotation system (46). A list of 389 ISGs was derived from previously published work (47). StringDB (48) and Cytoscape (49) were used in conjunction to generate an organically clustered interactome of functionally associated genes (StringDB, confidence >0.4). Edges represent a combination of significant ISG × ISG Pearson correlation and documented functional relationship as identified by StringDB (true for both criteria). Edge color represents the status of the ISG × ISG association following correction for *CHROMR* expression via partial correlation analysis.

### Cellular Response to Microbial Ligands and Influenza Infection.

To assess the response of macrophages to TLR activation and viral infection, we stimulated THP-1 macrophages or GapmeR-treated THP-1 macrophages with either 100–500 ng/mL LPS (Invivogen), 1 μg/mL poly(I:C) (Invivogen), IAV/WSN/1933 (H1N1), or vehicle control for indicated time periods. After treatment RNA was isolated and analyzed. Supernatants of GapCTRL-treated and Gap*CHROMR*-treated THP-1 stimulated for 24 h with 1 µg/mL of poly(I:C) were collected to measure accumulated levels of secreted cytokines. Levels of cytokines in supernatants were quantified with LEGENDplex Human Proinflammatory Cytokine Panel 1 (BioLegend, 740985) according to manufacturers’ instructions.

### ChIRP.

Cell harvesting, lysis, disruption, and chromatin isolation by RNA purification were performed as previously described (50), with modifications detailed in the *SI Appendix*, *SI Materials and Methods*. A list of probes used in this study can be found in *SI Appendix*, Table S6. DNA and protein were isolated from hybridized magnetic beads followed by DNA sequencing (ChIRP-seq) or ChiRP-MS as detailed below.

### ChIRP-seq.

Isolated ChIRP DNA was purified via PCR purification columns (Zymo Research) and subjected to Illumina sequencing. Reads were trimmed with Trimmomatic (51) and mapped to hg19 with BWA (52). Peaks were called for each probe set and replicate via the *callpeak* function from MACS2 (53) relative to the input from the same replicate. Peaks were imported into the DiffBind package from Bioconductor (54), and differential peaks were called between even (ChiRP_1) and odd (ChiRP_2) probe sets. Peaks with no differential binding between the probe sets were retained. Peaks were assigned to their nearest genomic location with the ChIPseeker package from Bioconductor (55). ChIRP-seq data are deposited in the Gene Expression Omnibus (accession no. GSE190413).

### ChIRP-MS.

ChIP purified proteins were pelleted and solubilized in Laemmli sample buffer (Invitrogen). Next, protein samples were size-separated in Bis-Tris sodium dodecyl sulfate–polyacrylamide gel electrophoresis gels (Invitrogen) and submitted for mass spectrometry analysis by the Proteomics Laboratory at NYU Langone Health. Only high-confidence peptides, based on a better than 1% false discovery rate searched against a decoy database, were included for peptide identification. Each protein was scored by the sum of the scores of the individual peptide sequences present. Mean score of three experiments was calculated as the average of the individual protein scores of each individual mass spectrometry experiment.

### Statistics.

Statistical significance between two groups of independent biological replicates was evaluated with Student’s *t* test. One-way ANOVA was performed when we compared three or more groups for one variable (univariate comparisons), followed if significant by either Dunnett’s post hoc multiple comparisons test (MCT) when we compared to a control group or Sidak’s post hoc MCT when we compared preselected groups. For nonparametric measurements, Kruskal–Wallis test was performed, followed by Dunn’s MCT. Repeated-measure two-way ANOVA was used to compare two or more groups for bivariate analyses followed by Sidak’s post hoc MCT to compare groups if either group or group × time interaction was significant.

Pearson correlation was used to examine ISG × ISG correlation in influenza A–infected patients, for which the sample size of *n* = 41 can detect a correlation of *r* = 0.43 with α = 0.05 and a power of 80%. Robust third-order polynomial nonlinear regression was used to assess distribution of lncRNA × ISG correlation coefficient in function of differential expression in influenza A infection to minimize outlier impact. RNA-seq normalized transcript data were log_1P_-transformed to normalize distribution for partial correlation analysis. Partial correlation analysis was used to control for *CHROMR* expression as a covariate within ISG × ISG associations. Pearson and partial correlation coefficients were compared by Fisher-*r*-to-*Z* transformation followed by *Z*-test.

Statistical significance of enrichment in top canonical pathways and top upstream regulators (cytokines and transcriptional regulators) was calculated in Ingenuity Pathway Analysis via right-tailed Fisher’s exact test. Enrichr, Genomic Regions Enrichment of Annotations Tool, and Hypergeometric Optimization of Motif EnRichment use a binomial test to calculate significant enrichment in biological process or motif enrichment, respectively. Statistical analyses were performed in GraphPad Prism software, and bivariate and partial correlation analyses were performed in RStudio. The threshold for statistical significance was *P* ≤ 0.05. All quantitative data are presented as mean ± SEM.

## Supplementary Material

Supplementary File

## Data Availability

RNA-seq, ChIP-seq, and ChIRP-seq datasets have been deposited in the Gene Expression Omnibus (GEO) and are available under accession number GSE190413 (56). All study data are included in the article or *SI Appendix*.
