# Supplementary material for: Long noncoding RNA CHROMR regulates antiviral immunity in humans
Source: Proc Natl Acad Sci U S A. 2022 Aug 24;119(37):e2210321119. doi: 10.1073/pnas.2210321119 (PMC9477407; doi:10.1073/pnas.2210321119)
Supplement: Supplementary File [file pnas.2210321119.sapp.pdf]

PNAS

Supplementary Information for

Long non-coding RNA *CHROMR* regulates antiviral immunity in humans

Coen van Solingen[1], Yannick Cyr[1], Kaitlyn R. Scacalossi[1], Maren de Vries[2], Tessa J. Barrett[1], Annika de Jong[1], Morgane Gourvest[1], Tracy Zhang[1], Daniel Peled[1], Raadhika Kher[1], MacIntosh Cornwell[3], Michael A. Gildea[1], Emily J. Brown[1], Stephanie Fanucchi[4], Musa M. Mhlanga[5,6,7], Jeffrey S. Berger[1], Meike Dittmann*[2], and Kathryn J. Moore*[1,8]

***Correspondence to:**

Kathryn J. Moore, PhD, NYU Cardiovascular Research Center, New York University Langone Health, 435 E 30th Street, Science Building 706, New York, NY 10016, USA. E-mail: kathryn.moore@nyulangone.org

Meike Dittmann, PhD, Department of Microbiology, New York University Langone Health, 430 E 29th Street, Alexandria Center for Life Sciences, West Tower, Room 313, New York, NY 10016, USA. E-mail: meike.dittmann@nyulangone.org

This PDF file includes:

Supplementary Material and Methods
Figures S1 to S5
Tables S1 to S6
Supplementary Index References

Supplementary Materials and Methods

Cell culture. *CHROMR* expression in human monocyte-derived macrophages infected with influenza A/California/04/09 (H1N1), influenza A/Wyoming/03/03 (H3N2), influenza A/Vietnam/1203/04 (H5N1) or mock-infected was examined by querying publicly available dataset GSE97672 (1).

HEK293T and THP-1 cell lines were obtained from ATCC and the NF-κB-SEAP and IRF-Lucia luciferase Reporter Monocytes (THP-1-Dual cells) were obtained from InvivoGen. All cell lines were authenticated using standard ATCC methods (morphology check by microscope, growth curve analysis) and tested monthly for mycoplasma contamination. HEK293T were maintained in high-glucose DMEM (Corning) supplemented with 10% fetal bovine serum (FBS, Life Technologies) and 1% penicillin/streptomycin (P/S, Life Technologies). THP-1 cells were maintained in RPMI 1640 (ATCC) supplemented with 10% FBS and 1% P/S. THP-1-Dual cells were maintained in RPMI 1640 supplemented 10% FBS, 1% P/S, and 50 μg/mL of Normocin (InvivoGen). THP-1-Dual cells were cultured with selectable marker Zeocin (100 μg/mL, InvivoGen) every other passage to maintain stable integration of inducible reporter constructs. THP-1 cells and THP-1-Dual Cells were differentiated into macrophages in the presence of 100 nM phorbol-12-myristate acetate (PMA, Sigma) for 48-72h.

Transient knockdown of *CHROMR* was acquired as follows; PMA-differentiated THP-1 cells or PMA-differentiated THP-1-Dual cells were transfected with 62.5 nM locked nucleic acid GapmeRs (Qiagen) targeting a common region of all *CHROMR* variants (Gap*CHROMR*) or Negative Control A (GapCTRL) using Lipofectamine RNAiMax (Life Technologies) as described (2). *CHROMR3*-overexpressing THP-1 cells were created as described (2), and cultured under selection pressure puromycin (5 μg/mL, Thermo Fisher Scientific) to maintain purity. Knockdown of *IRF2* and *IRF2BP2* was acquired by transfecting 100 nM siRNA directed against *IRF2* (Qiagen, GS3660) or *IRF2BP2* (Qiagen, GS359948) using Lipofectamine RNAiMax into THP-1 macrophages, Allstars Negative Control (Qiagen, 1027280) was used control.

RNA isolation, cell fractionation and qPCR. Total RNA was isolated using TRIzol reagent (Invitrogen) and Direct-zol RNA MicroPrep columns (Zymo Research). For cell fractionation experiments RNA was isolated from separate cytoplasmic and nuclear fractions using the PARIS kit (Thermo Fisher Scientific). Upon isolation, RNA was reverse transcribed using iScript cDNA Synthesis kit (Bio-Rad Laboratories) and quantitative PCR analysis was conducted using KAPA SYBR green Supermix (KAPA Biosystems) according to the manufacturer's instructions and quantified on Quantstudio 3 (Applied Biosystems). Fold change in mRNA expression was calculated using the comparative cycle method ($2^{-\Delta\Delta Ct}$) normalized to the housekeeping gene *GAPDH*. A list of primers used in this study can be found in ***SI Appendix,*** **Table S6**.

RNA-sequencing. RNA was isolated from THP-1 macrophages treated with *CHROMR*-targeting GapmeRs or negative control and subsequently stimulated with 500 ng/mL LPS or 1 µg/mL poly(I:C) (InvivoGen) for indicated times. RNA was used to generate barcoded cDNA libraries using the TruSeq RNA Sample Preparation kit (Illumina). Indexed libraries were pooled and sequenced (paired-end 50 or 100 bp reads) on the Illumina HiSEQ platform. RNA-seq reads were aligned using the STAR Aligner against hg38 annotations. Gene counting was done using featureCounts. Raw counts were normalized and DE-analysis was performed using DESeq2. Ingenuity Pathway Analysis (Qiagen) was used to evaluate the most significantly altered pathways and upstream regulators. RNA-seq data are deposited in the GEO under the accession number GSE190413.

Gene expression profiling. RNA was isolated from THP-1 macrophages stable overexpressing *CHROMR* or an empty vector control, reverse-transcribed, and qPCR analysis of Type I Interferon Response genes was performed using RT^2 Profiler PCR Arrays (Qiagen, PAHS-016ZA) according to manufacturer's protocol. Data analysis was performed using the manufacturer's integrated web-based software package of the PCR Array System using ΔΔCt-based fold change calculations.

Quantification of influenza A virus infection in THP-1 macrophages. THP-1 macrophages transiently knocked down for *CHROMR* were infected with 100, 500 or 1000 plaque forming units (PFU, as determined on MDCK cells) of influenza A/WSN/1933 (H1N1) virus. The virus inoculate was diluted in DPBS supplemented with calcium and magnesium. Cell growth media was replaced by virus dilution and incubated for 1h at 37°C and 5% CO_2. After 1h, the virus was aspirated, RPMI 1640 with 20% FBS was added to the cells, and cells were incubated at 37°C and 5% CO_2. At 24h, the cells were fixed with 8% paraformaldehyde (Thermo Fisher Scientific), quenched with 50 mM NH_4Cl and washed with PBS. Cells were stained with a monoclonal mouse anti-NP antibody (Sigma, MAB8251) followed by anti-mouse Alexa 488 secondary antibody (Thermo Fisher Scientific, R37120) and nuclear staining (4′,6-diamidino-2-phenylindole (DAPI, Sigma). Cells were washed with PBS leaving the last wash on before imaging. Plates were imaged using the Cell-Insight CX7 high-content screening platform. Images were analyzed and quantified with HCS Navigator software for total and infected cell numbers.

THP-1-Dual reporter assay. THP-1-Dual cells were differentiated towards macrophages using PMA and subsequently transfected with GapmeRs targeted *CHROMR* or a GapmeR control as described above, 24h post-transfection the THP-1-Dual cells were treated with 1 µg/mL poly(I:C). Supernatants were taken on indicated time points and activation of NF-κB was measured by detecting secreted alkaline phosphatase (SEAP) using Quanti-Blue (InvivoGen); activation of the

Interferon Regulatory Factor (IRF) at the ISRE was measured by detecting luciferase levels in the supernatants using Quanti-Luc (InvivoGen). Detected levels of SEAP and luciferase at the start of the experiment (0h) were set to 100%.

Chromatin immunoprecipitation. GapCTRL and Gap*CHROMR* treated THP-1 macrophages were cross-linked with 1% glutaraldehyde (Thermo Fisher Scientific) for 10 min at room temperature, and the cross-linking was quenched twice with 125 mM glycine/PBS for 5 min each. Cells were washed twice with ice-cold PBS, and harvested by scraping. Nuclear pellets were isolated by swelling cross-linked cells in hypotonic lysis buffer (25 mM HEPES pH 7.4, 1.5 mM $MgCl_2$, 10 mM KCl, 0.5% NP-40 and 1 mM DTT) supplemented with 1x HALT protease inhibitor cocktail (Promega) on ice for 15 min, followed by Dounce homogenization. Nuclear pellets were suspended in sonication buffer (50 mM HEPES pH 7.4, 140 mM NaCl, 1 mM EDTA, 1% Triton-X 100, 0.1% sodium deoxycholate, 0.5% SDS, 1 mM DTT and 1x protease inhibitor cocktail) and incubated at ice for 10 min. Nuclear extracts were sonicated using Bioruptor UCD-200 (Diagenode Inc.) for 10 x 1 min cycles of "30 sec ON / OFF" at the highest voltage setting to generate 200 - 500 bp chromatin fragments. In each experiment, chromatin was first processed by agarose gel electrophoresis to confirm DNA shearing to 200 - 500 bp fragments, and the DNA concentration was measured by NanoDrop 2000. Equal quantities of sheared chromatin (10 µg per immunoprecipitation) were diluted 1:5 in sonication buffer to the final volume of 1 mL, and immunoprecipitated overnight with 1 µg antibody targeting human histone H3K27Ac (Active Motif, 39685) or isotype control IgG antibody (Sigma, 12-370) at 4°C overnight. Chromatin complexes were captured using 20 µL Dynabeads protein G (Invitrogen) at 4°C for 1h. Beads were washed once with sonication buffer (containing 0.1% SDS), two times with high salt buffer (50 mM HEPES pH 7.4, 500 mM NaCl, 1 mM EDTA, 1% Triton-X 100, 0.1% sodium deoxycholate, 0.1% SDS), two times with LiCl buffer (20 mM Tris pH 7.4, 250 mM LiCl, 1 mM EDTA, 0.5% NP-40, 0.1% sodium deoxycholate, 0.05% Tween-20), and once with Tris-EDTA buffer (10 mM Tris pH 7.4, 1 mM EDTA). Each wash was performed at room temperature for 5 min in 1 mL volume. Beads were captured using DynaMag magnet (Thermo Fisher Scientific). Elution was performed by suspending beads in 100 µL elution buffer (20 mM Tris pH 7.4, 1% SDS, 50 mM $NaHCO_3$, 1 mM EDTA). Chromatin immunoprecipitation (ChIP) eluates were reverse cross-linked at 65°C for 4h, digested with proteinase K (Thermo Fisher Scientific, 10 µg/mL) at 55°C for 1h and 2 µL RNase cocktail (Ambion) at 37°C for 30 min.

ChIP-sequencing. ChIP purified DNA was cleaned using PCR purification columns (Qiagen) and subjected to Illumina sequencing. Next, the overall quality of the sequenced ChIP-seq libraries was assessed with FastQC (3). To remove contaminating sequencing adapters and low-quality bases, reads were trimmed using Fastp (4). FastQC was run again on the trimmed reads to analyze the

global impact of trimming. Reads were then aligned to the human genome (hg38) using Bowtie2 (5). Alignments were sorted and indexed using Samtools for downstream processes (6). MACS2 was then used to identify significant peaks (7). Peaks with a Q-value of less than 0.05 were retained. Custom scripts and the ChIPQC R package were used to assess ChIP-seq peak quality and reproducibility (8). Peaks present in all replicates from each condition were retained for differential enrichment analysis. Peaks were annotated using ChIPseeker package from Bioconductor (9). Peaks that overlapped a 4kb window centered at an annotated transcription start site were annotated as promoter peaks. Differential enrichment analysis was performed using DiffBind package from Bioconductor (10). Peaks with a false discovery rate (FDR-)adjusted P-value of 0.1 or less were considered differentially enriched between the knockdown and control conditions. Functional analysis of significantly enriched peaks was performed using Genomic Regions Enrichment Annotations Tool (GREAT) with default parameters (11). Motif enrichment analysis was performed on 3kb windows centered at the TSS of genes with differentially enriched promoter peaks using Hypergeometric Optimization of Motif EnRichment (HOMER) with the command '*findMotifs.pl*' (12). ChIP-seq data are deposited in the GEO under the accession number GSE190413.

Chromatin Isolation by RNA Precipitation (ChIRP). Cell harvesting, lysis, disruption, and chromatin isolation by RNA purification were performed as previously described (13) with the following modifications: 1) Cells were cross-linked in 3% formaldehyde for 30 min, followed by 0.125 M glycine quenching for 5 min; 2) Hybridization was performed for 16h; 3) For mass spectrometry (MS) experiments, lysates were pre-cleared by incubating with 30 mL washed beads per mL of lysate at 37°C for 30 min with mixing; 4) As a negative control, lysates were pooled and aliquoted into equal amounts and RNA was removed by incubating with RNase A (1 µg/mL, Sigma), and subsequent incubation at 37°C for 30 min prior to hybridization steps. RNA, DNA, protein isolation was performed as described (13) and further detailed below for ChIRP followed by DNA-seq (ChIRP-seq) or Comprehensive Identification of RNA-binding Proteins by Mass Spectrometry (ChIRP-MS). RNA extraction was performed for validation of lncRNA enrichment. A list of probes used in this study can be found in ***SI Appendix*, Table S6**.

ChIRP followed by DNA-seq (ChIRP-seq). DNA was eluted from hybridized magnetic beads and subjected for Illumina sequencing. In short, beads were washed at room temperature with ChIRP wash buffer (EMD Millipore, #17-10494). Beads were subsequently captured using a DynaMag magnet (Thermo Fisher Scientific) and DNA was eluted by suspending beads in elution buffer (20 mM Tris pH 7.4, 1% SDS, 50 mM NaHCO3, 1 mM EDTA). ChIRP eluates were reverse cross-linked at 65°C for 4h, digested with Proteinase K (EMD Millipore) at 55°C followed by incubation with RNase cocktail (Ambion). ChIRP purified DNA was cleaned using PCR purification columns

(Zymo Research) and subjected to Illumina sequencing. Reads were trimmed using Trimmomatic (14) and mapped to hg19 using BWA (15). Peaks were then called for each probe set and replicate using the '*callpeak*' function from MACS2 (7) relative to the input from the same replicate. Peaks were then imported into the DiffBind package from Bioconductor (10) and differential peaks were called between even and odd probe sets. Only peaks with no differential binding between the probe sets were retained. Peaks were then assigned to their nearest genomic location using ChIPseeker package from Bioconductor (9). ChIRP-seq data are deposited in the GEO under the accession number GSE190413.

Comprehensive Identification of RNA-binding Proteins by Mass Spectrometry (ChIRP-MS). Protein was isolated from magnetic beads and analyzed by MS. To elute protein beads were collected on magnetic stand, resuspended in biotin elution buffer (12.5 mM D-biotin (Thermo Fisher Scientific), 7.5 mM HEPES pH 7.5, 75 mM NaCl, 1.5 mM EDTA, 0.15% SDS, 0.075% sarkosyl, and 0.02% sodium deoxycholate). Trichloroacetic acid (25% of total volume) was added to the clean eluent and proteins were precipitated at 4°C overnight. Proteins were pelleted at 16,000 g at 4°C for 30 min, washed with cold acetone and pelleted again at 16,000 g at 4°C for 5 min. Proteins were immediately solubilized in desired volumes of Laemmli sample buffer (Invitrogen) and boiled at 95°C for 30 min with occasional mixing to reverse crosslinking. Final protein samples were size-separated in Bis-Tris SDS-PAGE gels (Invitrogen) and submitted for MS analysis by the Proteomics Laboratory at NYU Langone Health. Individual samples were subjected to liquid chromatography (LC) separation with MS using the autosampler of an EASY-nLC 1000 (Thermo Fisher Scientific). Subsequently, peptides were gradient eluted from the column directly to Q Exactive mass spectrometer using a 1h gradient (Thermo Fisher Scientific). High resolution full MS spectra were acquired with a resolution of 70,000, an AGC target of 1×10^6, with a maximum ion time of 120 ms, and scan range of 400 to 1,500 m/z. Following each full MS twenty data-dependent high resolution HCD MS/MS spectra were acquired. All MS/MS spectra were collected using the following instrument parameters: resolution of 17,500, AGC target of 5×10^4, maximum ion time of 120 ms, one microscan, 2 m/z isolation window, fixed first mass of 150 m/z, and NCE of 27. MS/MS spectra were searched against a UniProt human database, using Sequest (16) within Proteome Discoverer (Thermo Fisher Scientific). Only high confidence peptides, based on a better than 1% FDR searched against a decoy database, were included for peptide identification. Each protein was scored by the sum of the scores of the individual peptide sequences present. Mean score of three experiments is calculated as the average of the individual protein scores of each individual MS-experiment.

RNA Immunoprecipitation. Human histone H3, IRF2BP2, and HNRPNLL were immunoprecipitated from PMA-differentiated THP-1 macrophages. All immunoprecipitations were

done using the MagnaRIP RNA-Binding Protein Immunoprecipitation Kit (EMD Millipore) according to the manufacturers' instructions. Briefly, an antibody targeting human histone H3 (Abcam, ab1791), IRF2BP2 (Abcam, ab220155), HNRNPLL (Cell Signaling, 4783), or an isotype matched control antibody (Sigma, 12-370 or 12-371) were bound to magnetic beads and incubated with lysed cells at 4°C for 24h. Beads were isolated and cleaved from the bound proteins by proteinase K, and coprecipitated RNA was purified. qPCR analysis of total RNA was performed to detect enrichment of *CHROMR* variants and control genes in the protein-of-interest precipitated fraction was determined as percentage of 1% input control.

RNA Fluorescence *In Situ* Hybridization. Custom Stellaris® FISH Probes were designed against *CHROMR* utilizing the Stellaris® FISH Probe Designer (LGC Biosearch Technologies). Formaldehyde-fixed THP-1 macrophages were permeabilized with 70% isopropanol and subsequently simultaneously hybridized with the *CHROMR* Stellaris® FISH Probe set labeled with Quasar® 670 Dye (LGC Biosearch Technologies) and a rabbit polyclonal antibody against IRF2BP2 (Atlas Antibodies, HPA062269), following the manufacturer's protocol. IRF2BP2 was visualized using fluorescent goat anti-rabbit secondary antibodies (Thermo Fisher Scientific, A-21206) and DAPI was used to visualize nuclear DNA.

Mutagenesis studies. The interaction between IRF2BP2 and *CHROMR3* was analysed by mutating the putative interaction site between IRF2BP2 and *CHROMR3*. Two GG-doublets in the sequence of a plasmid overexpressing *CHROMR3* (2) were replaced with two CC-doublets creating a plasmid overexpressing *CHROMR3*-G4mut. All mutations were performed using the Quickchange XL kit (Stratagene) using the primers indicated in ***SI Appendix,*** **Table S6**. *CHROMR3*-G4mut expression plasmids were confirmed by Sanger sequencing. Next, human IRF2BP2, *CHROMR3* and *CHROMR3*-G4mut were overexpressed in HEK293T cells using plasmids overexpressing a MYC/DDK tagged IRF2BP2 (OriGene Technologies, RC213250) and plasmids overexpressing *CHROMR3* and *CHROMR3*-G4mut using Lipofectamine 2000 (Thermo Fisher Scientific). Antibodies directed against MYC/DDK (OriGene Technologies, TA50011) or an isotype matched control antibody (Sigma, 12-370) were used in immunoprecipitations as described above.

Bioinformatics. Enrichment analysis of interferon-stimulated genes was performed using Enrichr (17), the web-based software for Gene Set Enrichment Analysis was used for ChIP Enrichment Analysis (ChEA) (18) database (2016) which contains results from transcription factor ChIP-seq studies extracted from supporting material. Results were manually curated to remove duplicate studies or studies performed with non-human transcription factors. The catRAPID algorithm (19) was used to estimate the binding propensity of IRF2BP2 and *CHROMR*. The interaction score is

generated using the interaction propensity distribution of a reference set, as described (20). The QGRS Mapper (21) was used for recognition and mapping of putative quadruplexes in *CHROMR*. RNAfold, part of The Vienna RNA Websuite (22), was used to predict the minimum free energy secondary structure of *CHROMR3* and the RNA plot was created with RNArtist, developed by Fabrice Jossinet and available at https://github.com/fjossinet/RNArtist.

Figure S1

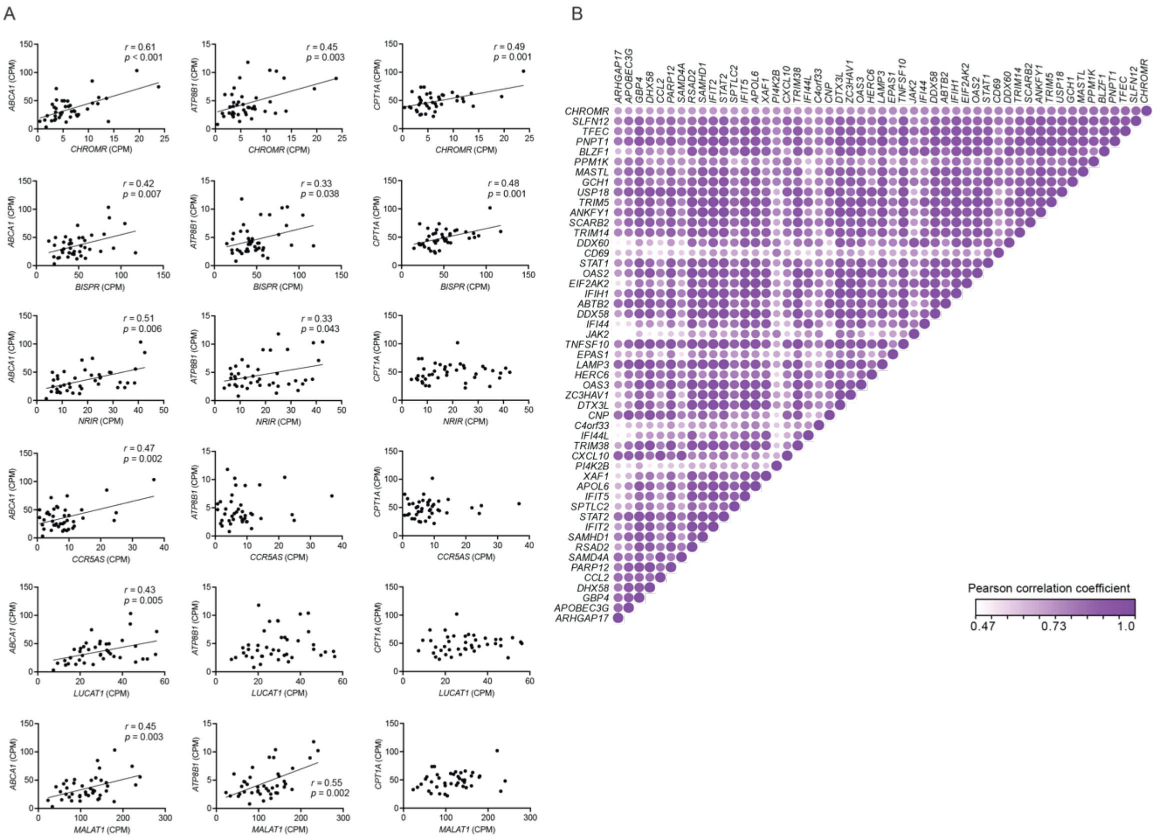

***CHROMR* expression levels in whole blood of patients infected with influenza A virus is associated with expression of cholesterol efflux genes and interferon-stimulated genes**. (A) Plots showing correlation coefficient between lncRNAs and cholesterol efflux genes *ABCA1*, *ATP8B1*, *and CPT1A* expressed in whole blood of patients with influenza A virus. (B) Pearson correlation matrix showing the 50 ISGs that are most strongly associated with *CHROMR* in whole blood in IAV-infected patients. (A) r = Pearson correlation coefficient, (B) Individual dot size and color represent Pearson correlation coefficient and absence of dot indicate lack of association. P < 0.05.

Figure S2

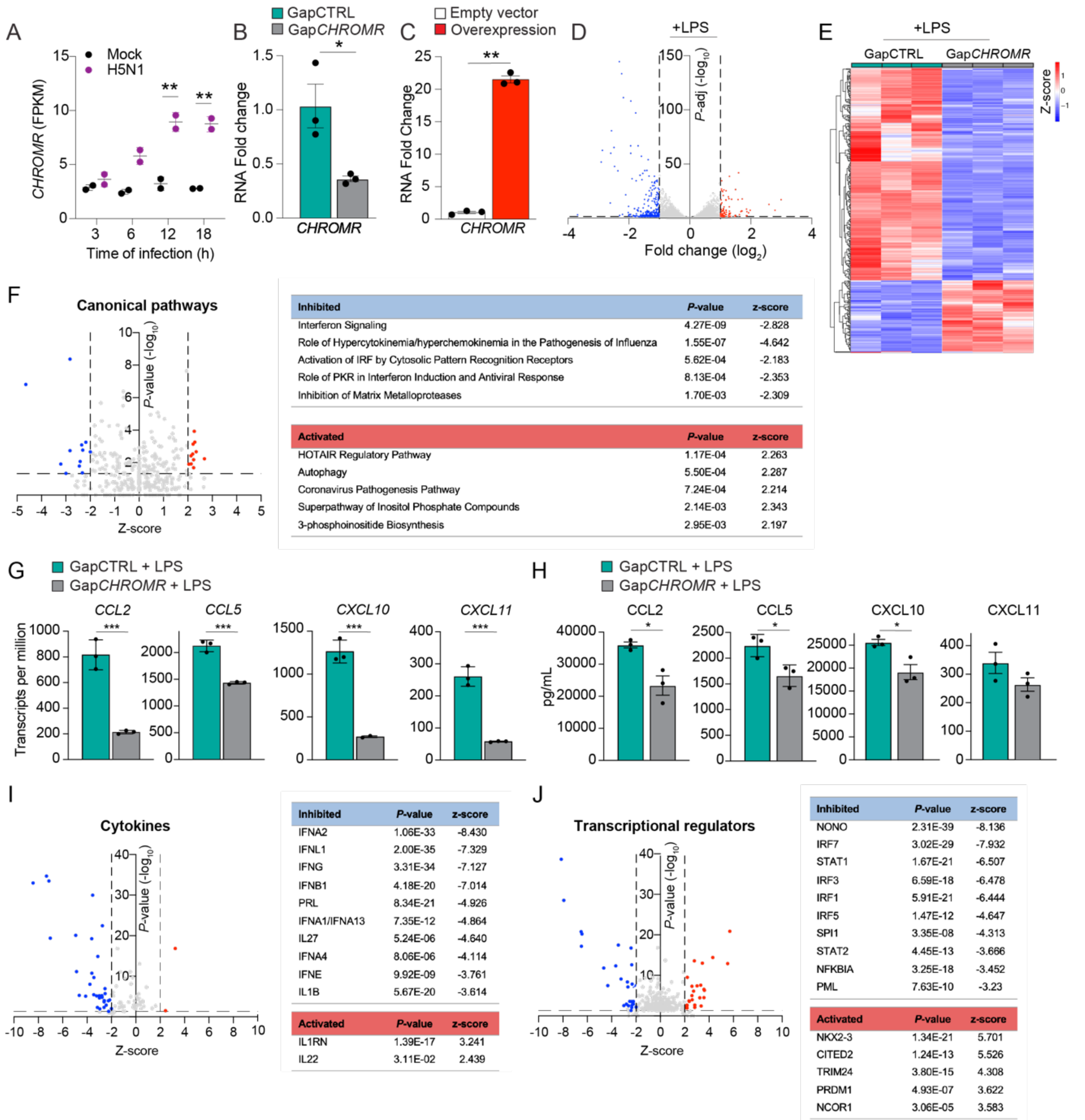

Inhibited	P-value	z-score
Interferon Signaling	4.27E-09	-2.828
Role of Hypercytokinemia/hyperchemokinemia in the Pathogenesis of Influenza	1.55E-07	-4.642
Activation of IRF by Cytosolic Pattern Recognition Receptors	5.62E-04	-2.183
Role of PKR in Interferon Induction and Antiviral Response	8.13E-04	-2.353
Inhibition of Matrix Metalloproteases	1.70E-03	-2.309

Activated	P-value	z-score
HOTAIR Regulatory Pathway	1.17E-04	2.263
Autophagy	5.50E-04	2.287
Coronavirus Pathogenesis Pathway	7.24E-04	2.214
Superpathway of Inositol Phosphate Compounds	2.14E-03	2.343
3-phosphoinositide Biosynthesis	2.95E-03	2.197

Inhibited	P-value	z-score
IFNA2	1.06E-33	-8.430
IFNL1	2.00E-35	-7.329
IFNG	3.31E-34	-7.127
IFNB1	4.18E-20	-7.014
PRL	8.34E-21	-4.926
IFNA1/IFNA13	7.35E-12	-4.864
IL27	5.24E-06	-4.640
IFNA4	8.06E-06	-4.114
IFNE	9.92E-09	-3.761
IL1B	5.67E-20	-3.614

Activated	P-value	z-score
IL1RN	1.39E-17	3.241
IL22	3.11E-02	2.439

Inhibited	P-value	z-score
NONO	2.31E-39	-8.136
IRF7	3.02E-29	-7.932
STAT1	1.67E-21	-6.507
IRF3	6.59E-18	-6.478
IRF1	5.91E-21	-6.444
IRF5	1.47E-12	-4.647
SPI1	3.35E-08	-4.313
STAT2	4.45E-13	-3.666
NFKBIA	3.25E-18	-3.452
PML	7.63E-10	-3.23

Activated	P-value	z-score
NKX2-3	1.34E-21	5.701
CITED2	1.24E-13	5.526
TRIM24	3.80E-15	4.308
PRDM1	4.93E-07	3.622
NCOR1	3.06E-05	3.583

***CHROMR* is required for LPS induction of interferon-stimulated gene expression**. (A) *CHROMR* expression (FKPM) in human monocyte-derived macrophages infected with influenza A/Vietnam/1203/2004 (H5N1), or mock infected. (B-C) qPCR analysis of *CHROMR* in THP-1 macrophages after transfection with *CHROMR*-targeting (Gap*CHROMR*) and control GapmeRs (GapCTRL) (B) or in THP-1 macrophages stable overexpressing *CHROMR* or an empty vector control (C). (D) Volcano plot showing differential expression of genes identified by RNA-seq of THP-1 macrophages transfected with Gap*CHROMR* versus GapCTRL and stimulated with LPS (500 ng/mL) for 3h. Dashed lines indicate fold change (log2) = +/-1; P-adj = 0.05; red dots indicate upregulated genes; blue dots indicate downregulated genes; grey dots indicate non-significantly changed genes. (E) Hierarchical clustering heatmap showing normalized gene expression values

in THP-1 macrophages transfected with Gap*CHROMR* or GapCTRL and subsequently stimulated with LPS (500 ng/mL) for 3h. Cutoffs used for visualization: -2 < FC > 2; and P-adj < 0.05. (F, I and J) Volcano plots and tables of most affected canonical pathways (F), cytokines (I), and transcriptional regulators (J) identified through Ingenuity Pathway Analysis of (E); dashed lines indicate Z-score = +/- 2 and P-adj = 0.05; red dots indicate upregulated factors and blue dots indicate downregulated factors; non-significantly changed factors are indicated by grey dots. (G-H) Normalized expression counts (G) and protein levels (H) for CCL2, CCL5, CXCL10, and CXCL11 in THP-1 macrophages transfected with Gap*CHROMR* or GapCTRL and subsequently stimulated with LPS (100 ng/mL) for 3h (G) or 24h (H). Data are mean +/- standard error of the mean for 2 (A) or 3 (D to G, I and J) independent experiments, or representative of 3 independent experiments (B, C and H). P values were calculated using a repeated measures two-way ANOVA with Sidak's multiple comparison test (A), right-tailed Fisher's exact test (F, I and J), or a two-tailed unpaired Student's t-test (B, C, G and H). *P < 0.05; **P < 0.01; ***P < 0.001.

Figure S3

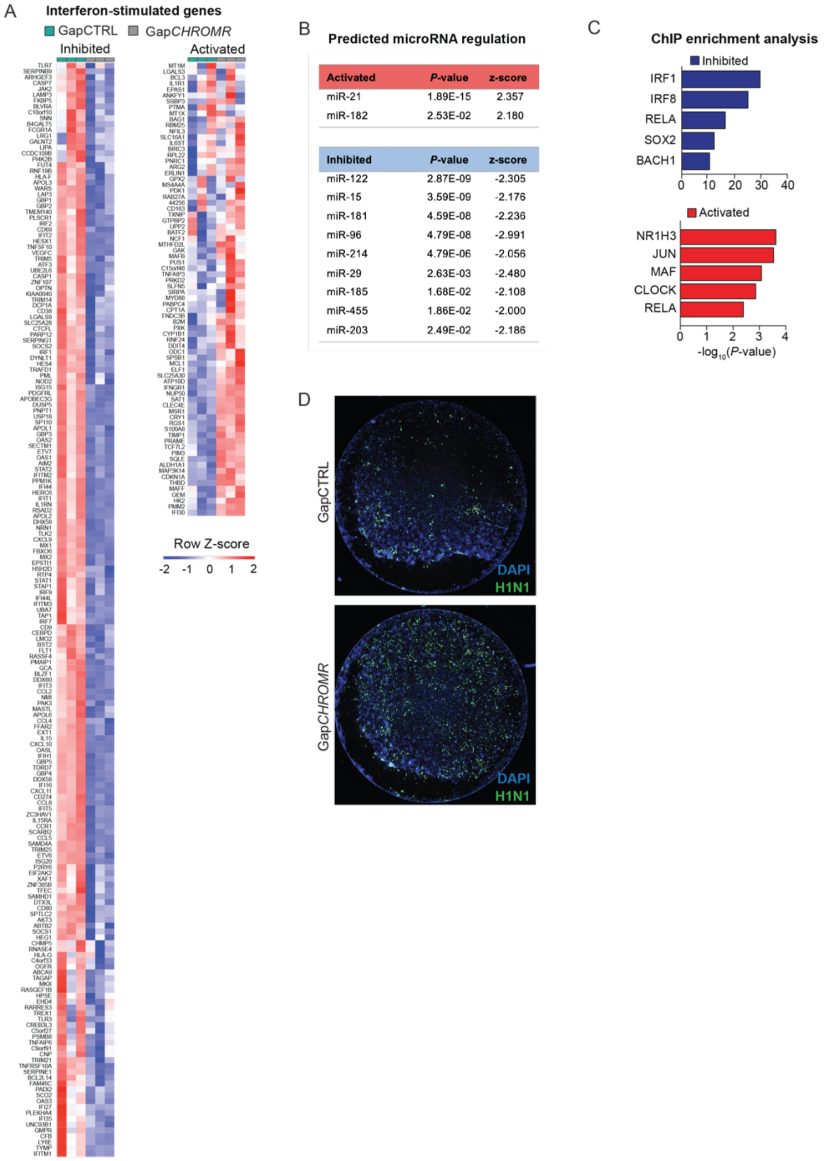

Activated	P-value	z-score
miR-21	1.89E-15	2.357
miR-182	2.53E-02	2.180

Inhibited	P-value	z-score
miR-122	2.87E-09	-2.305
miR-15	3.59E-09	-2.176
miR-181	4.59E-08	-2.236
miR-96	4.79E-08	-2.991
miR-214	4.79E-06	-2.056
miR-29	2.63E-03	-2.480
miR-185	1.68E-02	-2.108
miR-455	1.86E-02	-2.000
miR-203	2.49E-02	-2.186

***CHROMR* regulates ISG expression in response to LPS.** (A) Hierarchical clustering heatmap showing Z-scores of differentially expressed interferon-stimulated genes in THP-1 macrophages transfected with Gap*CHROMR* or GapCTRL and subsequently treated with LPS (500 ng/mL) for 3h, P-adj<0.05. (B) List of most affected microRNAs identified through Ingenuity Pathway Analysis of Fig. 2C. (C) Bar plots showing transcription factor binding enrichment analysis against the ChIP Enrichment Analysis (ChEA 2016) database gene set library using the inhibited genes (top) and activated genes (bottom) indicated in Fig. S3A. (D) Representative whole-well microscopy images of immunofluorescent staining for influenza-A-infected (H1N1, green) THP-1 macrophages transfected with GapCTRL or Gap*CHROMR* and counterstained for nuclear RNA (DAPI, blue). P values were calculated using Binomial test (B-C).

Figure S4

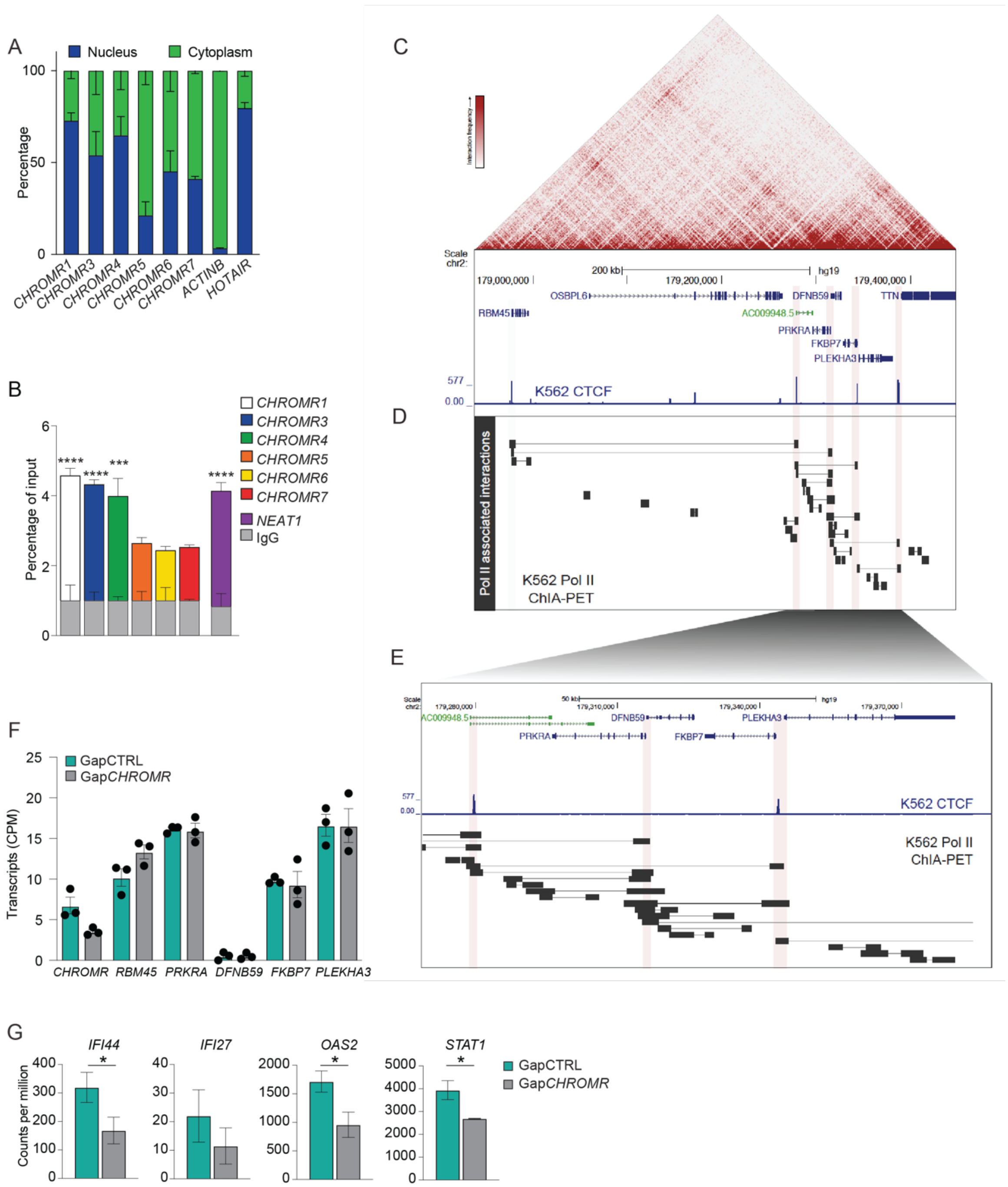

***CHROMR* is enriched in the nucleus and interacts with histones.** (A) qPCR analysis of *CHROMR* variants after cellular fractionation of THP-1 macrophages. *ActinB* and lncRNA *HOTAIR* are used as cytoplasmic and nuclear controls, respectively. (B) Enrichment of *CHROMR* variants in H3-immunoprecipitates from THP-1 macrophages relative to IgG control. lncRNA *NEAT1* was used as H3-enriched control. (C) Heatmap showing chromatin interactions at genomic location of *CHROMR*. (D) Pol II ChIA-PET analysis in K562 indicating chromatin interactions within genomic

location indicated in (C) black, connected bars indicate direct interactions. (E) Zoom of topologically associated domain (TAD) containing *CHROMR*. Connected bars indicate direct interactions. (F) Normalized transcript reads (CPM) of genes present in *CHROMR*'s TAD indicated in (D and E) from THP-1 macrophages transfected Gap*CHROMR* or GapCTRL. (G) Normalized counts of ISGs in unstimulated THP-1 cells treated with Gap*CHROMR* or GapCTRL. Data (A, B, F, G) are mean +/- standard error of the mean for 3 independent experiments. P values were calculated using a repeated measures two-way ANOVA with Sidak's multiple comparison test (B) or a Student's t-test (G). *P < 0.05, ***P < 0.001; ****P < 0.0001.

Figure S5

A

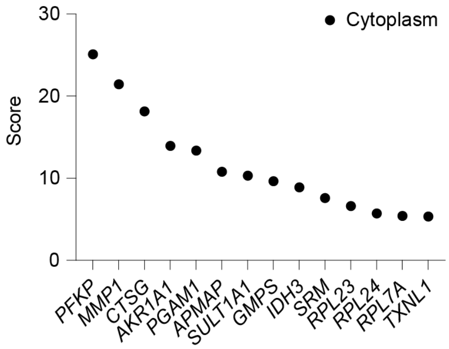

B

QGRS sequences found (overlaps not included)

Position	Length	QGRS	G-Score
15	30	GGCACAAAAAGCAGGAACGAGTAGCGGTGG	11
237	28	GGGGGAGGTCCCAAAGCGGATATCCTGG	16
675	29	GGCTGGTCTCAAACTTTTGGCCTCAAGGG	11

C

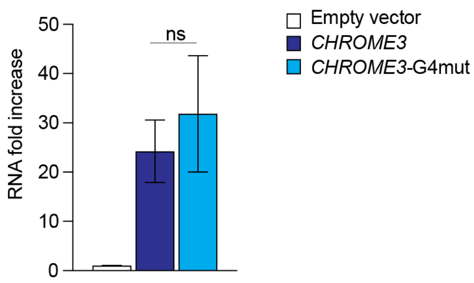

(A) Identification of cytoplasmic *CHROMR*-binding proteins by ChIRP-MS (Comprehensive identification of RNA-binding proteins by mass spectrometry) in THP-1 macrophages. Mean score of 3 independent experiments. (B) QGRS-mapper analysis of putative Quadruplex forming G-Rich Sequences (QGRS, G-quadruplex) in *CHROME3*. Putative G-quadruplexes are highlighted in blue. (C) qPCR analysis of *CHROME3* expression in HEK- 293T cells transfected with plasmids expressing *CHROME3*, *CHROME3*-G4mut or control. Data are mean +/- standard error of the mean for 2 independent experiments (C). P values were calculated using a Student's t-test (C).

Table S1. Demographics of COVID-19 Whole Blood RNA-Seq cohort

	**Control**	**SARS-CoV-2**
**n**	7	8
**Age, median (IQR)**	61 (59, 66)	64 (58, 68)
**Sex**		
Female, n (%)	2 (29)	3 (38)
**Race**		
Non-White, n (%)	4 (57)	7 (88)
Hispanic, n (%)	4 (57)	3 (38)
**Comorbidities**		
BMI, median (IQR)	26.9 (24.7, 28.6)	27.6 (24.8, 31.5)
Diabetes, n (%)	1 (14)	5 (71)
Hyperlipidemia, n (%)	4 (57)	2 (25)
Hypertension, n (%)	3 (43)	5 (63)
**Hospitalization**		
Mechanical ventilation, n (%)	0 (0)	7 (88)
Discharged, n (%)	7 (100)	3 (38)
Ongoing, n (%)*	0 (0)	1 (13)
Deceased, n (%)	0 (0)	4 (50)

*Patient status at time of whole blood RNA sequencing

Table S2. LncRNAs differentially expressed in both influenza A and SarsCoV2 infection

	INFLUENZA A		SARS-COV2	
LncRNA	log2FoldChange	padj	log2FoldChange	padj
A2M-AS1	-0.97	6.48E-03	7.54	2.93E-13
AATBC	1.37	1.04E-12	6.39	1.47E-12
ABALON	1.01	5.92E-03	8.56	2.78E-23
ADAMTSL4-AS1	1.73	8.00E-12	7.43	2.68E-18
ALMS1-IT1	-0.84	4.33E-02	4.79	1.23E-03
AOAH-IT1	1.68	3.28E-08	4.25	4.02E-04
ARMCX5-GPRASP2	-1.07	9.15E-03	5.83	8.72E-09
ATP11A-AS1	0.89	9.90E-03	7.33	6.49E-15
BASP1-AS1	1.29	6.46E-03	4.46	3.23E-06
BISPR	1.85	1.80E-22	6.31	2.60E-18
C1QTNF7-AS1	0.96	4.49E-02	4.4	3.41E-05
C5orf67	1.86	1.21E-06	4.5	3.87E-04
CAHM	0.9	3.00E-04	4.42	1.33E-05
CARD8-AS1	0.84	1.04E-08	6.46	4.21E-52
CCR5AS	2.92	1.53E-23	4.8	5.77E-14
CEROX1	-0.85	8.56E-05	3.56	2.93E-02
CFAP58-DT	2.5	4.05E-19	7.26	3.01E-26
CFLAR-AS1	1.14	1.54E-04	7.42	1.99E-28
CHROMR	1.88	1.58E-10	5.76	4.57E-20
CKMT2-AS1	-0.66	1.95E-02	5.48	2.05E-08
CLRN1-AS1	1.27	4.21E-02	4.67	6.03E-04
CYTOR	1.35	2.16E-12	5.95	1.35E-40
DHRS4-AS1	-0.71	5.39E-07	7.56	1.48E-20
DIAPH1-AS1	1.02	2.60E-02	7.2	4.65E-15
DISC1-IT1	2.78	1.04E-09	5.92	1.52E-05
DLEU2	1.1	1.20E-07	6.64	1.02E-87
DOCK4-AS1	2.54	8.47E-07	5.04	1.64E-05
DOCK8-AS1	1.31	2.94E-06	8.49	6.96E-55
DPYD-AS1	1.11	4.21E-02	6.36	3.08E-10
ECE1-AS1	0.59	1.92E-02	6.43	5.13E-12
EIF1B-AS1	0.71	2.06E-02	7.16	9.94E-19
EPB41L4A-AS1	-0.64	8.31E-05	5.54	1.26E-21
ERICH6-AS1	-1.07	5.56E-04	5.04	2.70E-07
FAM157C	0.77	1.20E-03	7.55	1.81E-17
FAM198B-AS1	0.94	5.11E-04	6.69	1.44E-13
FOXN3-AS1	0.91	2.94E-04	4.43	6.49E-06
FRY-AS1	-0.89	1.10E-02	5.16	1.49E-07
GABPB1-AS1	-0.74	3.34E-04	7.96	8.95E-37

GAS5	-0.69	1.11E-05	3.56	9.74E-22
GSEC	1.6	2.19E-07	8.94	3.37E-22
GSN-AS1	2.03	4.79E-10	7.51	2.38E-16
HCG27	0.74	1.25E-05	9.99	2.74E-35
HCP5	0.61	7.84E-09	6.99	8.32E-101
HEIH	0.65	7.58E-04	3.85	2.89E-17
HIF1A-AS3	1.25	3.49E-03	6.7	2.54E-14
HLA-F-AS1	0.88	8.15E-05	6.54	6.05E-14
HOTAIRM1	0.72	6.52E-05	8.55	7.90E-23
HOXB-AS1	-1.37	6.47E-07	3.02	1.05E-02
ID2-AS1	0.77	1.26E-02	4.24	9.97E-04
IRF1-AS1	1.9	2.99E-18	9.15	1.68E-37
ITPK1-AS1	0.76	4.89E-03	8.15	8.51E-30
JPX	1.33	2.57E-10	7.54	1.83E-23
KCNJ2-AS1	1.24	1.74E-04	5.98	3.61E-13
KLF3-AS1	-1.11	2.98E-06	2.96	8.07E-04
L3MBTL2-AS1	0.75	1.49E-02	4.92	2.40E-06
LBX2-AS1	0.94	1.17E-04	4.17	9.63E-06
LINC00189	1.29	1.24E-03	5.24	2.01E-06
LINC00205	-0.8	5.52E-04	6.76	3.21E-15
LINC00243	-0.69	4.23E-03	7.12	1.74E-15
LINC00342	-0.78	1.47E-03	7.04	6.24E-23
LINC00467	0.6	4.85E-02	6.45	5.56E-14
LINC00482	1.36	9.71E-13	5.49	4.69E-08
LINC00526	-0.88	6.69E-03	4.51	2.15E-04
LINC00539	-0.69	2.59E-02	6.89	2.33E-13
LINC00570	1.1	2.95E-03	9.47	1.62E-29
LINC00654	0.78	1.62E-02	4.07	4.09E-05
LINC00663	-0.71	8.78E-04	4.98	2.16E-05
LINC00665	-0.83	1.90E-02	3.89	6.58E-04
LINC00667	-0.64	5.33E-09	6.16	2.37E-41
LINC00847	0.75	1.51E-04	6.67	9.80E-13
LINC00852	-0.65	1.16E-02	4.52	3.43E-04
LINC00853	0.96	4.42E-03	6.28	2.79E-12
LINC00861	-0.78	1.80E-06	9.35	2.88E-31
LINC00877	0.94	4.79E-05	7.44	8.14E-36
LINC00885	1.11	4.37E-02	4.25	3.87E-04
LINC00921	-0.7	1.67E-08	7.51	1.91E-18
LINC00926	-0.86	1.27E-03	6.25	7.41E-09
LINC00957	-0.78	6.26E-08	5.18	1.06E-07
LINC00968	1.76	6.43E-07	5.24	9.58E-07
LINC00989	0.84	1.43E-02	8.3	5.28E-20
LINC01006	-1.02	1.27E-04	3.96	1.09E-04

LINC01011	1	4.50E-05	6.92	1.77E-13
LINC01089	-0.87	3.39E-10	6.21	5.79E-12
LINC01093	3.45	7.99E-13	6.54	1.41E-08
LINC01094	2.14	1.52E-10	5.54	9.07E-22
LINC01138	0.76	1.06E-07	8.83	5.87E-25
LINC01146	1.14	4.83E-03	5.65	8.44E-09
LINC01176	0.71	3.11E-03	6.68	4.68E-11
LINC01184	-0.72	1.53E-07	6.05	2.03E-16
LINC01191	0.76	2.02E-02	4.65	1.51E-05
LINC01215	-1.26	7.46E-07	6.53	2.79E-11
LINC01232	0.78	8.89E-04	7.3	6.77E-15
LINC01270	0.87	4.19E-03	6.51	1.27E-15
LINC01303	1.28	5.49E-05	4.34	6.19E-08
LINC01311	-0.74	9.89E-04	4.11	3.37E-04
LINC01353	1.34	7.61E-05	5.35	6.92E-08
LINC01359	1.38	5.43E-05	6.82	9.30E-14
LINC01410	1.45	5.84E-16	9.53	1.03E-31
LINC01485	1.36	3.27E-03	5.25	2.56E-06
LINC01504	1.54	2.43E-13	7.17	7.32E-16
LINC01506	1.53	6.14E-08	10.7	1.26E-37
LINC01531	2.57	1.69E-16	7.33	7.38E-15
LINC01550	-1.28	7.20E-09	7.23	1.95E-10
LINC01619	-1.06	6.25E-03	4.95	9.99E-05
LINC01637	0.79	9.54E-03	4.83	3.93E-07
LINC01762	1.55	1.68E-05	5.63	2.01E-09
LINC01963	-0.61	5.12E-03	6.07	3.42E-11
LINC02009	1.36	2.51E-03	6.9	8.69E-08
LINC02035	-0.79	6.03E-06	7.69	2.42E-24
LINC02158	-0.94	2.54E-03	7.53	1.32E-17
LINC02212	1.32	2.34E-04	4.01	2.57E-03
LINC02213	1.09	2.23E-02	7.32	2.36E-18
LINC02217	1.48	2.79E-03	7.51	1.18E-14
LINC02273	-0.86	9.75E-04	6.92	7.78E-12
LINC02325	-0.69	2.56E-02	4.72	1.70E-04
LINC02352	0.62	2.95E-03	9.14	4.47E-30
LINC02362	0.95	1.18E-02	6.24	7.35E-15
LINC02363	1.34	8.96E-05	4.89	1.67E-07
LINC02384	1	1.08E-03	7.31	3.51E-16
LINC02397	-0.62	3.21E-02	6.09	1.31E-06
LINC02422	1.88	8.08E-14	8.51	3.02E-25
LINC02432	1.14	2.53E-03	6.95	2.14E-13
LINC02446	-1.04	3.28E-03	7.06	1.23E-09
LINC02470	1.13	3.61E-02	8.26	6.67E-08

LINC02471	3.42	9.24E-16	6.98	1.64E-12
LINC02528	3.96	1.62E-20	3.77	1.77E-02
LINC02611	-0.64	1.11E-03	6.17	9.15E-09
LINC02648	-1.05	1.07E-03	4.27	7.89E-04
LINC02649	0.96	2.24E-03	6.73	2.27E-14
LINC02656	1.28	3.26E-04	6.39	3.45E-12
LINC02724	0.91	7.53E-04	5.28	1.25E-08
LINC02762	-0.76	3.67E-02	7.06	2.32E-17
LOXL1-AS1	-0.84	2.47E-03	5.96	2.49E-09
LUNAR1	-1.34	1.09E-02	6.28	1.19E-09
MALINC1	-0.74	4.32E-02	5.29	1.92E-06
MAP3K14-AS1	-0.69	1.26E-03	4.63	7.53E-07
MAP3K5-AS1	1.12	2.11E-03	4.9	1.80E-06
MDS2	-1.36	3.45E-08	5.24	2.58E-05
MHENCR	-1	1.40E-10	7.36	1.12E-18
MIR155HG	-0.89	3.13E-02	1.18	1.45E-02
MIR3142HG	-0.67	3.35E-02	2.22	3.86E-04
MIR3945HG	2.76	2.06E-16	3.06	6.52E-09
MIR4435-2HG	1.36	4.67E-15	6.41	9.42E-55
MIR9-3HG	-1.19	3.51E-06	3.52	1.54E-02
MIRLET7BHG	-0.63	1.76E-05	6.09	8.07E-12
MKNK1-AS1	0.71	2.45E-02	3.98	7.34E-03
NAPA-AS1	0.76	9.39E-04	4.99	8.54E-07
NEAT1	0.87	3.61E-05	8.44	1.01E-93
NRIR	3.35	4.87E-22	7.5	1.73E-15
OLMALINC	-1.21	1.77E-05	4.9	6.93E-06
PCBP1-AS1	1.58	4.92E-13	8.17	2.65E-47
PIK3CD-AS1	0.74	8.62E-06	7.37	2.34E-19
PINK1-AS	-0.7	3.18E-04	5.92	3.66E-11
PKN2-AS1	2.5	1.52E-10	3.34	3.79E-04
PPP1R12A-AS1	1.28	3.53E-03	4.59	2.25E-05
PRKAG2-AS1	-0.94	7.74E-04	4.87	9.01E-06
PRKCQ-AS1	-0.71	6.12E-04	8.72	9.98E-23
PSMB8-AS1	0.64	1.92E-06	8.49	8.99E-71
PSMD6-AS2	0.75	4.48E-04	7.25	9.83E-16
PSMG3-AS1	-0.64	3.04E-03	4.93	5.34E-07
RAD51-AS1	-0.89	6.49E-05	6.85	1.97E-18
RARA-AS1	0.99	3.11E-08	7.85	3.75E-30
RERE-AS1	1.62	2.61E-11	8	5.21E-21
RNASEH2B-AS1	1.52	7.46E-04	5.28	2.66E-07
RNF213-AS1	2.27	1.39E-16	5.72	7.94E-13
SATB1-AS1	-1.19	6.80E-04	5.11	2.68E-07
SCAMP1-AS1	1.3	3.25E-07	5.93	1.52E-13

SCARNA9	0.64	7.90E-03	7.34	6.56E-18
SENCR	0.86	7.87E-06	5.75	2.24E-13
SERPINB9P1	1.79	1.52E-08	1.92	4.13E-04
SLC25A25-AS1	-0.87	1.20E-07	4.18	8.92E-06
SNHG14	-0.76	9.79E-03	5.89	3.35E-20
SNHG25	-1.07	8.36E-03	4.48	6.06E-05
SNHG29	-0.84	4.62E-11	5.53	4.80E-47
SNHG5	-0.69	1.55E-03	6.47	1.14E-72
SNHG8	-0.86	2.04E-06	6.26	2.96E-24
SNHG9	-0.73	6.36E-04	6.73	9.50E-14
ST20-AS1	-0.66	5.22E-04	6.48	3.19E-14
TET2-AS1	0.94	3.66E-02	7.21	8.72E-17
TNK2-AS1	1.57	7.97E-12	6	3.07E-11
TPRG1-AS1	1.32	7.45E-04	5.89	1.58E-09
TRHDE-AS1	1.27	4.24E-02	5.7	2.23E-08
TRIM52-AS1	-0.8	3.25E-05	7.91	2.66E-20
TSPOAP1-AS1	-0.71	6.60E-09	6.81	7.62E-16
TTC28-AS1	-0.64	1.37E-04	7.26	1.95E-17
TTN-AS1	1.02	9.50E-04	9.03	1.46E-31
UGDH-AS1	-0.81	3.53E-02	4.59	2.19E-04
USP30-AS1	1.31	3.77E-11	5.12	5.24E-06
VIPR1-AS1	-1.45	2.30E-04	4.85	7.87E-07
ZNF667-AS1	-0.91	6.84E-03	4.76	1.45E-07

Table S3. Genes differentially expressed in THP-1 macrophages treated with Gap*CHROMR* versus GapCTRL after poly(I:C) stimulation for 8h.

Gene	log2FoldChange	padj
EGR1	3.31	8.14E-54
STAT1	-1.18	7.94E-41
MS4A7	-1.67	1.84E-39
IL10RA	-2.06	5.02E-37
MX2	-1.44	3.40E-36
JAG1	1.56	3.85E-35
TOB1	-1.65	4.27E-35
IFI44L	-2.41	1.03E-33
TIMP3	1.08	2.21E-33
PARP9	-1.39	9.64E-33
TNFSF14	1.83	2.62E-32
TRIB1	1.57	6.09E-32
CXCL3	1.34	2.65E-31
SLC2A5	-1.3	5.62E-31
OAS3	-1	5.62E-31
SLC20A1	1.17	1.05E-30
PARP14	-1.34	2.45E-30
GPRIN3	-1.92	1.37E-29
DUSP10	1.1	1.66E-29
SIGLEC1	-1.14	4.60E-29
EPSTI1	-1.31	1.84E-28
S1PR3	1.77	2.59E-28
RGMB	1.97	2.72E-28
ECE1	1.5	3.34E-28
XAF1	-1.64	3.66E-28
SAMD9L	-1.69	3.92E-28
TRIM22	-1.47	1.77E-27
SPRED2	1.18	2.59E-27
SLCO4A1	1.36	2.74E-27
SAMHD1	-1.35	4.30E-27
MX1	-1.45	1.23E-26
GPR68	1.69	2.76E-26
REL	-1.21	7.00E-26
SAMD9	-1.23	7.16E-25
OAS2	-1.06	7.16E-25
TRIM14	-1.18	2.47E-24
ONECUT2	-3.25	1.27E-23
DNAJB5	1.31	2.24E-23
POU2F2	1.23	4.44E-23

FGFRL1	1.14	5.62E-23
CITED4	1.76	5.79E-23
NTSR1	1.5	9.63E-23
STX1A	1.63	1.61E-22
MAF	-1.5	1.97E-22
BMP2K	-1.07	2.00E-22
RCBTB2	-1.53	2.73E-22
ITGA3	1.05	4.11E-22
GDF15	2.04	4.42E-22
DUSP5	1.49	1.02E-21
HMGB3	-1.24	1.61E-21
MS4A14	-1.67	2.30E-21
GBP1	-1.76	2.61E-21
ZSWIM4	1.31	4.99E-21
SIGLEC15	1.29	5.93E-21
EIF2AK2	-1.15	7.01E-21
NRIP3	1.35	1.13E-20
MERTK	-1.68	1.71E-20
PIM1	1.61	2.42E-20
CD36	-1.41	2.66E-20
SLC43A2	-1.29	2.66E-20
NPC1	1.21	3.22E-20
ITGA5	1.01	3.26E-20
LINC01094	-2.42	5.31E-20
THBD	1.17	5.58E-20
ITPRIP	1.18	6.41E-20
BHLHE41	1.29	7.46E-20
AREG	2.35	8.23E-20
DDX60	-1.26	8.35E-20
COL6A3	1.28	9.40E-20
MAFF	1.48	9.49E-20
SPHK1	1.42	9.54E-20
TET2	-1.05	1.40E-19
S100A9	-1.24	2.81E-19
CORO2A	-1.74	3.06E-19
EMP1	1.43	6.20E-19
SIK1	1.37	7.15E-19
DAPP1	-1.81	8.82E-19
GPR183	-1.8	3.18E-18
RUSC2	1.09	4.14E-18
PLK3	1.37	4.31E-18
MAP1S	1.09	4.49E-18
SMTN	1.32	5.62E-18

UCN2	1.21	6.92E-18
IFIH1	-1.19	8.61E-18
IL36RN	2.49	9.06E-18
MARCH1	-1.77	9.83E-18
IRF2BPL	1.11	1.69E-17
PLSCR1	-1.08	4.20E-17
KDM6B	1.22	4.20E-17
PTPRC	-1.36	1.14E-16
GLUL	-2.69	1.16E-16
MITF	-1.25	2.48E-16
SLA	-1.17	4.15E-16
FAM13A	-1.88	4.91E-16
FGD4	-2.31	5.99E-16
PHLDA2	1.8	6.60E-16
IRAK3	-1.34	6.63E-16
RASA3	1.01	7.85E-16
IRS2	1.13	7.85E-16
PEX5L	-1.52	8.85E-16
KCNN4	1.18	1.22E-15
RBL1	-1.38	1.24E-15
PLXDC2	-1.45	1.34E-15
C9orf66	-1.32	1.54E-15
FGR	1.02	1.54E-15
TREM2	-1.29	1.76E-15
ADORA2B	1.79	1.76E-15
BCL6	1.06	2.70E-15
SIRPB2	-3.19	3.41E-15
BRCA2	-1.37	5.52E-15
CLEC7A	-3.08	6.64E-15
TLR4	-1.46	6.72E-15
GUCY1A2	-1.39	8.79E-15
LIF	2.46	8.88E-15
LPAR6	-1.41	9.41E-15
S100A16	1.29	1.15E-14
ZNF107	-1.59	1.28E-14
MOB3A	1.08	1.31E-14
S100A8	-1.6	1.58E-14
TNFSF10	-2.15	3.94E-14
APBB1IP	-1.54	4.11E-14
SLC39A14	1.03	4.47E-14
ICAM5	1.05	5.93E-14
IFNGR1	-1.01	6.26E-14
PTPRO	-1.9	6.85E-14

NRSN1	2.56	6.90E-14
STK40	1.13	7.15E-14
C10orf54	-1.39	7.23E-14
NRIP1	-1.09	7.23E-14
RHBDF1	1.43	7.42E-14
STARD8	1.19	7.66E-14
E2F7	1.04	8.62E-14
STX3	1.09	9.65E-14
MAP2K3	1.3	1.68E-13
PDE4D	-1.76	1.79E-13
HERC6	-1.18	1.79E-13
DACT1	1.85	2.14E-13
FRY	-1	2.20E-13
FCAR	1.23	3.43E-13
IFI44	-1.25	4.69E-13
CMPK2	-1.17	5.31E-13
FCGR3A	-1.74	5.96E-13
LRRC8C	-1.26	7.31E-13
GFPT2	1.38	7.32E-13
UBASH3B	-1.26	8.06E-13
OAF	1.22	8.77E-13
PTPRE	1.04	1.16E-12
LOC100294362	-1.38	1.22E-12
TMCC3	2.13	1.26E-12
IER2	1.15	1.59E-12
SERPINB8	1.03	1.67E-12
ICOSLG	-1.21	1.73E-12
PDGFRB	1.54	1.74E-12
CENPF	-1.19	1.84E-12
JDP2	-1.71	2.25E-12
TLDC2	-1.14	2.50E-12
FYB	-1.96	3.05E-12
TOMM34	1.03	3.16E-12
FILIP1L	-1.17	4.70E-12
LDLR	1.71	5.17E-12
MMS22L	-1.41	5.57E-12
GBP4	-1.44	6.67E-12
SASH3	-1.29	6.68E-12
LINC00941	1.75	6.81E-12
RHOU	-1.47	7.17E-12
RARG	1.09	7.17E-12
CYP27A1	-1.24	7.25E-12
HS3ST3A1	1.26	7.25E-12

GAB2	-1.17	7.68E-12
MGAT4A	-1.32	8.01E-12
POLQ	-1.54	1.01E-11
SNX2	-1.07	1.02E-11
DOK2	-1.56	1.15E-11
IPCEF1	-1.33	1.35E-11
HOPX	1.09	1.67E-11
CLSPN	-1.46	1.76E-11
ALOX5	-1.5	1.79E-11
ME1	-1.13	1.93E-11
ISG15	-1.29	2.98E-11
TOP2A	-1.16	3.51E-11
MNDA	-3.09	5.20E-11
FZD5	-2.02	7.27E-11
TMEM200A	1.24	7.50E-11
GNG11	1.26	8.10E-11
FAM105A	-2.56	9.34E-11
MYEOV	1.57	9.34E-11
PCDH18	-1.63	1.02E-10
TNFRSF10D	1.24	1.19E-10
ATAD2	-1.1	1.86E-10
SH3RF1	1.03	2.10E-10
ADAM28	-1.15	2.31E-10
ESCO2	-1.42	2.38E-10
DMWD	1.02	2.65E-10
SMC4	-1.05	2.73E-10
CCNE2	-1.51	3.18E-10
OASL	-1.1	3.50E-10
DGKH	-1.1	4.51E-10
SERPINE2	1.14	5.54E-10
SMAGP	2.08	5.84E-10
CORO2B	1.47	5.88E-10
DLL1	1.58	5.97E-10
SPRED3	1.87	7.35E-10
DTL	-1.29	7.42E-10
SNAI1	-1.19	7.66E-10
HELLS	-2.15	9.07E-10
KL	-1.73	1.25E-09
TMEM170B	-1.38	1.31E-09
NLRC4	-1.41	1.41E-09
THSD7A	-1.47	1.47E-09
ADRBK2	-1.62	1.62E-09
CCL3L1	3.45	1.63E-09

STIL	-1.04	1.71E-09
TNFSF13B	-1.91	1.86E-09
EREG	1.61	1.97E-09
BRCA1	-1.22	1.98E-09
POLA1	-1.37	2.08E-09
TM4SF19-TCTEX1D2	1.76	2.60E-09
GALM	-1.34	2.64E-09
SLC8A1	-1.24	3.01E-09
CXorf21	-1.54	3.03E-09
TREML1	-1.34	3.08E-09
INTS7	-1.02	3.16E-09
ZNF93	-1.66	3.19E-09
CCL3L3	3.4	3.70E-09
FCRLB	1.18	3.91E-09
ETS2	-1.05	4.37E-09
GBP5	-1.06	4.50E-09
MATK	1.05	4.79E-09
FAM111B	-1.81	6.21E-09
METTL7B	1.19	6.30E-09
ARHGEF40	-1.1	7.16E-09
TBX21	3.5	8.01E-09
TM4SF19	1.78	8.82E-09
KRT79	1.41	1.02E-08
MYRF	1.08	1.03E-08
STX17	-1.08	1.13E-08
MFSD2A	1.67	1.29E-08
GCNT1	-2.03	1.34E-08
MAST4	1.21	1.34E-08
KNTC1	-1.08	1.57E-08
IL7R	-2.08	1.62E-08
GAB3	-1.41	1.73E-08
MNT	1.1	1.93E-08
S100A12	-2.66	2.01E-08
ASPM	-1.12	2.03E-08
FAM102A	1.01	2.18E-08
ARHGAP23	1.04	2.18E-08
PMP22	1.85	2.23E-08
NUSAP1	-1.11	2.30E-08
MLPH	1.48	3.05E-08
RAD54B	-1.55	3.32E-08
TM4SF1	1.6	3.50E-08
NCAPG	-1.16	3.74E-08
EDNRB	-1.92	3.93E-08

PKIB	-1.11	4.13E-08
PRRX1	1.27	4.65E-08
IL36B	1.5	4.83E-08
NCOA2	-1.3	4.91E-08
P2RY6	-1.11	5.19E-08
CD86	-2.14	5.39E-08
SNX30	-1.08	5.79E-08
CHIC2	1.44	6.13E-08
NAV2	1.76	6.31E-08
LRRK2	-1.91	6.35E-08
CITED2	1.75	6.52E-08
SLC2A3	1.26	6.69E-08
TBX15	-1.61	6.83E-08
ADCY7	-1.13	6.94E-08
UPP1	1.41	7.83E-08
LOC100506585	-2.04	8.67E-08
JAK2	-1.06	8.69E-08
ZMIZ1-AS1	1.01	8.83E-08
CEP152	-1.47	9.08E-08
SOX13	2.04	9.09E-08
TM4SF19-AS1	1.85	9.45E-08
OLR1	-1.72	1.01E-07
PLXNA2	1.77	1.08E-07
BRCC3	-1.14	1.20E-07
CNTLN	-1.15	1.41E-07
ATAD5	-1.24	1.43E-07
PAX8-AS1	1.29	1.53E-07
GPR176	1.01	1.54E-07
CDC42EP3	1.12	1.70E-07
TRIB2	1.28	1.83E-07
USP45	-1.16	2.19E-07
XKRX	1.65	2.19E-07
MLH3	-1.07	2.30E-07
RASSF2	-2.27	2.69E-07
IFIT3	-1.95	2.69E-07
CD84	-1.39	2.71E-07
FAM13A-AS1	-1.9	3.09E-07
RTTN	-1.34	3.20E-07
DCBLD2	1.09	3.37E-07
TCF7L2	1.18	4.23E-07
EXO1	-1.12	4.47E-07
SDC1	1.36	4.61E-07
ARPIN	-1.18	4.82E-07

SRPX	1.19	5.06E-07
E2F8	-1.4	5.28E-07
IL1RN	1.56	5.30E-07
CYSLTR1	-1.34	5.63E-07
FAM109A	1.07	5.68E-07
USP18	-1.08	6.27E-07
TSC22D1	1.97	6.79E-07
ZNF525	-1.33	7.07E-07
GPR65	-1.03	7.68E-07
RMI1	-1.02	8.31E-07
AVPI1	-1.74	8.46E-07
LAMA4	1.56	8.54E-07
EMR3	-1.73	8.66E-07
MMP19	1.41	1.01E-06
SH2B3	1.49	1.02E-06
RGCC	1.57	1.06E-06
ZC3H12D	-1	1.08E-06
SPECC1	-1.39	1.11E-06
WDR76	-1.02	1.11E-06
ZNF480	-1.01	1.15E-06
FAT4	-1.17	1.16E-06
MCM10	-1.45	1.19E-06
FRMD6	1.37	1.19E-06
SLC25A37	1.06	1.20E-06
FBLIM1	1.72	1.35E-06
NOS2	2.79	1.40E-06
RASAL1	-2.64	1.54E-06
PAPPA-AS1	1.4	1.72E-06
PRL	-4.21	1.75E-06
SESN3	-1.55	1.75E-06
GPR85	-3.11	1.84E-06
HERC5	-1.31	1.84E-06
MKI67	-1.41	1.89E-06
WWTR1	1.59	1.97E-06
CDH2	1.34	2.01E-06
PTPRF	-1.2	2.07E-06
RBBP8	-1.28	2.11E-06
TFPI2	1.52	2.14E-06
DYNC2H1	-1.01	2.26E-06
CALD1	1.49	2.31E-06
CENPE	-1.27	2.40E-06
COL22A1	1.98	2.55E-06
TGFBI	-1.33	2.69E-06

PPARG	-1.01	3.01E-06
AIM1	-1.01	3.07E-06
GPRC5C	1.29	3.15E-06
BUB1	-1.08	3.49E-06
ZNF366	-1.56	3.58E-06
ZGRF1	-1.22	3.62E-06
ZNF780B	-1.22	4.17E-06
TYMS	-1.1	4.26E-06
SHCBP1	-1.05	4.28E-06
CTH	1.77	4.28E-06
DOT1L	1.29	4.35E-06
ANKRD45	-2.19	4.72E-06
SNCB	1.72	5.05E-06
KIF23	-1.16	5.41E-06
TEC	-1.23	5.59E-06
LOC100288637	-1.39	5.70E-06
STOX2	-1.39	5.89E-06
EHD1	1.37	5.91E-06
PLAUR	1.87	6.10E-06
RASSF4	-1.13	6.32E-06
FZD4	1.56	6.64E-06
LOXL2	1.06	7.77E-06
IFIT1	-1.73	7.82E-06
BLM	-1.23	7.83E-06
GCH1	1.4	7.83E-06
PTGS2	1.79	7.86E-06
RASGRP3	-1.05	8.09E-06
KIAA0101	-1.3	8.56E-06
AMY2B	-1.7	9.12E-06
CDCA2	-1.15	9.12E-06
ETS1	1.27	9.73E-06
DDX58	-1.56	1.02E-05
USP53	1.05	1.06E-05
FMN1	-1.06	1.14E-05
CACNA2D3	1.44	1.17E-05
HIST1H4I	-1.07	1.19E-05
NR1D1	1.42	1.20E-05
SCIMP	-3.06	1.22E-05
ABI3	-2.3	1.25E-05
RGS16	2.2	1.28E-05
CENPU	-1.12	1.29E-05
ATOH8	-2.32	1.34E-05
LAT	1.31	1.34E-05

LYRM7	-1.16	1.35E-05
CSF3	1.72	1.39E-05
ANTXR1	-1.11	1.41E-05
SPRY4	2.79	1.46E-05
CEP78	-1.21	1.50E-05
DIRAS2	1.95	1.59E-05
RAB39A	-1.16	1.62E-05
PTRF	1.06	1.72E-05
VIPR2	2.37	1.72E-05
KIF11	-1.21	1.80E-05
PPP1R15A	1.92	2.12E-05
FKBP10	1.36	2.15E-05
LOC729083	-1.81	2.17E-05
MDGA1	1.73	2.18E-05
GRIN3A	-2.04	2.23E-05
HIST2H3D	-1.15	2.26E-05
BUB1B	-1.13	2.26E-05
TLR8	-2.45	2.45E-05
CEMIP	1.01	2.45E-05
RPLP0P2	1.08	2.45E-05
ENOX1	-1.06	2.67E-05
TIGD2	-1.82	2.69E-05
RAP1GAP	1.01	2.74E-05
HBEGF	1.75	2.92E-05
MYBL1	-1.88	3.00E-05
SSC4D	-1.08	3.11E-05
LOC153684	-1.45	3.24E-05
SDS	-2.3	3.34E-05
PDE4B	-1.22	3.35E-05
CCDC144NL	-1.03	3.37E-05
IRGQ	1.18	3.38E-05
KIF21B	2.09	3.38E-05
ABCB10	-1.13	3.39E-05
LINC00460	2.34	3.49E-05
IFIT2	-2.13	3.62E-05
E2F2	-1.62	3.67E-05
SPDL1	-1.01	3.72E-05
PTP4A3	1.23	3.80E-05
PRSS35	-2.65	3.96E-05
TMEM255A	-1.11	4.01E-05
SEMA3G	-1.98	4.05E-05
SERPINE1	1.48	4.38E-05
NFATC2	1.11	4.51E-05

MMP3	1.93	4.51E-05
GEM	1.86	4.59E-05
PLK4	-1.08	4.79E-05
FOSL1	1.57	5.23E-05
ZNF100	-1.25	5.34E-05
FOSB	2.83	5.35E-05
APCDD1L	1.46	6.15E-05
HTR7	1.09	6.20E-05
SAMSN1	-1.95	6.37E-05
ULK2	-1.13	6.38E-05
PLD6	1.19	6.46E-05
RAB27A	-1.03	6.61E-05
CCL3	2.16	6.77E-05
CDR1	1.06	7.14E-05
GPR3	1.65	7.55E-05
ADORA2A	1.16	8.12E-05
CDC7	-1.24	8.15E-05
HPCAL1	1.16	8.17E-05
TSIX	1.84	8.45E-05
DUSP7	1.18	8.62E-05
IRAK2	1.58	8.96E-05
LINC01410	-1.5	9.13E-05
IL1B	2.93	9.15E-05
DCLRE1C	-1.13	9.32E-05
MPEG1	-1.9	9.81E-05
GPR56	1.48	1.02E-04
SPRY2	2.19	1.05E-04
HGD	-1.43	1.09E-04
TYMSOS	-1.46	1.10E-04
ZNF431	-1.19	1.14E-04
TNFSF15	2.16	1.16E-04
CEP128	-1.41	1.27E-04
CSF1	1.83	1.27E-04
GDAP1	-1.56	1.40E-04
POU4F2	1.05	1.46E-04
RSAD2	-1.9	1.54E-04
RNASE2	-1.03	1.61E-04
CXCL8	1.51	1.66E-04
KLHL29	-1.01	1.68E-04
KANK1	1.27	1.69E-04
SGOL1	-1.06	1.79E-04
CHAF1B	-1.1	1.84E-04
TBX3	1.21	1.85E-04

FES	-1.17	2.04E-04
DYNLL1-AS1	1.07	2.04E-04
TCHH	-1.59	2.06E-04
RGS5	-1.51	2.09E-04
PRIM1	-1	2.19E-04
KIF15	-1.21	2.29E-04
PTAFR	-1.19	2.31E-04
GPX3	1.19	2.31E-04
OSMR	1.04	2.38E-04
CYBB	-1.92	2.50E-04
FPR1	-4.73	2.54E-04
FGD2	-1.81	2.61E-04
NT5E	1.01	2.65E-04
GJA1	1.13	2.65E-04
PKDCC	-1.92	2.66E-04
ZNF678	-1.01	2.66E-04
BAALCOS	1.62	2.77E-04
FLNC	1.09	2.87E-04
MEG3	1.62	2.92E-04
RTP4	-1	2.98E-04
DDIT4L	2	3.10E-04
SORL1	-2.09	3.14E-04
MAS1	2.27	3.15E-04
ZNF33A	-1.39	3.16E-04
UBALD2	1.35	3.27E-04
TRHDE-AS1	-1.03	3.43E-04
PRSS22	2.49	3.52E-04
IL33	1.23	3.53E-04
KDELC2	-1.01	3.66E-04
PCDH1	1.4	3.68E-04
C5orf42	-1.18	3.84E-04
TSPAN13	1.22	3.84E-04
SNX9	1.56	3.88E-04
CSRNP1	1.14	3.96E-04
NR2F1	1.45	4.07E-04
ECM2	-2.39	4.39E-04
KIAA1524	-1.01	4.44E-04
POSTN	1.41	4.54E-04
SKA1	-1.13	4.61E-04
HIST1H4A	-1.85	4.64E-04
CCDC80	1.35	4.68E-04
FCGR2C	-1.98	4.71E-04
C5	-1.19	4.74E-04

LILRB2	-2.64	4.83E-04
WTAPP1	1.48	4.83E-04
TMEM86A	-1.1	4.86E-04
SEMA7A	1.47	5.05E-04
LOC100507065	1.54	5.14E-04
NEDD4L	1.56	5.16E-04
XIRP1	1.89	5.34E-04
NOD1	-1.04	5.35E-04
ZNF43	-1.42	5.46E-04
LINC01050	1.12	5.78E-04
RASL12	2.58	5.96E-04
PPARGC1A	-2.4	6.00E-04
COLEC12	-2.02	6.18E-04
ZNF273	-1.12	6.30E-04
HIST1H2BI	-1.93	6.41E-04
ODF3B	-1.41	6.49E-04
PHLDA1	1.25	6.77E-04
KLRK1	3.82	7.09E-04
LBH	1.1	7.12E-04
F2RL1	1.17	7.24E-04
HIST1H2AM	-1.02	7.27E-04
SGMS1	1.04	7.28E-04
LOC728084	1.33	7.48E-04
ZNF467	-1.9	7.49E-04
CEBPA	-1.11	7.57E-04
RGMB-AS1	1.69	7.79E-04
ELTD1	1.74	7.87E-04
RBM41	-1.1	7.88E-04
DRAXIN	2.61	8.02E-04
NEURL1B	-1.66	8.27E-04
CXCL10	-2.67	8.75E-04
DUSP6	1.15	8.81E-04
B3GNT5	-1.42	9.16E-04
ITGB3	1.46	9.34E-04
KLRC4-KLRK1	3.6	9.68E-04
DKK3	1.44	9.80E-04
ZNF138	-1.39	9.84E-04
ZNF675	-1.02	1.02E-03
ARNT2	1.01	1.04E-03
KCNQ3	-1.27	1.11E-03
SECTM1	-1.37	1.11E-03
SLC16A6	1.22	1.12E-03
KIAA0513	-1.27	1.12E-03

POLE2	-1.12	1.14E-03
SLC15A2	-1.19	1.15E-03
IRX3	1	1.19E-03
RAD51AP1	-1.73	1.21E-03
COL24A1	-1.18	1.21E-03
STAT4	-1.95	1.33E-03
KRT78	1.41	1.36E-03
COL5A2	1.09	1.37E-03
FOXP4	-1.35	1.37E-03
APBB2	1.09	1.39E-03
FMO5	-1.68	1.40E-03
ZNF441	-1.41	1.42E-03
SEC31B	-1.51	1.42E-03
SRC	1.34	1.44E-03
UPF3B	-1	1.45E-03
UCHL1	1.41	1.47E-03
HMGA2	2.01	1.48E-03
PDGFA	1.52	1.48E-03
HS3ST3B1	1.38	1.49E-03
CD101	-2.55	1.49E-03
FZD8	1.24	1.54E-03
AKAP12	1.09	1.57E-03
EFNB2	2.06	1.58E-03
KLF15	-2.27	1.59E-03
CRISPLD2	-1.11	1.64E-03
DUSP4	1.26	1.69E-03
DLC1	1.44	1.72E-03
SERPINA9	1.57	1.80E-03
AEBP1	1.21	1.88E-03
HIST4H4	-2.18	1.89E-03
MMP1	1.44	1.92E-03
XPA	-1.19	1.98E-03
GPCPD1	-1	2.04E-03
STC1	2.52	2.07E-03
C18orf54	-1.42	2.15E-03
RCSD1	-1.01	2.25E-03
SQLE	1.34	2.31E-03
KIAA2022	-2.4	2.36E-03
ZNF577	-1.11	2.40E-03
IL1A	1.15	2.42E-03
SENCR	-1.3	2.43E-03
STARD4	1.1	2.59E-03
DSEL	1.51	2.60E-03

CCND1	1.18	2.62E-03
ROBO4	1.11	2.74E-03
LOC284454	1.04	2.78E-03
LINC01204	2.34	2.80E-03
ZNF85	-1.2	2.84E-03
LAMB2	1.07	2.95E-03
ADAMTS12	1.31	2.96E-03
CBR3	-1.32	3.03E-03
KCNF1	2.23	3.08E-03
TUBA3FP	-1.6	3.09E-03
RGS4	1.13	3.19E-03
SPTA1	-1.62	3.22E-03
NUMB	1.03	3.24E-03
CENPI	-1.59	3.25E-03
VCAN	-1.1	3.30E-03
RNF125	-1.51	3.41E-03
LOC100272217	1.16	3.44E-03
LIPG	1.39	3.70E-03
TP73	-1.16	3.70E-03
CYP7B1	-1.12	3.75E-03
LARP6	1.41	3.89E-03
MYCT1	1.5	3.92E-03
CKMT2	-2.27	3.93E-03
ALOX12P2	-1.9	4.13E-03
KIAA1549L	1.7	4.22E-03
PCDHB4	-3.39	4.29E-03
CCL8	-2.76	4.40E-03
FAM83G	1.09	4.41E-03
FA2H	1.61	4.42E-03
SAMD3	1.45	4.45E-03
LRRC17	1.3	4.49E-03
FPR2	-2.5	4.61E-03
PTGIS	1.4	4.63E-03
ADORA3	-1.52	4.64E-03
C12orf75	1.24	4.65E-03
QRICH2	1.25	4.67E-03
ASPN	-2.26	4.78E-03
NGF	2.9	4.81E-03
OLFML2B	-1.18	4.88E-03
PDGFRA	1.55	5.11E-03
F2R	1.06	5.14E-03
FSBP	-1.71	5.17E-03
IL24	1.49	5.18E-03

LOC102724323	-2.47	5.25E-03
DLGAP1-AS2	1.55	5.25E-03
RPSAP52	1.99	5.28E-03
C9orf47	1.05	5.30E-03
FRMPD2	1.95	5.31E-03
OXER1	-1.79	5.32E-03
CCDC15	-1.18	5.37E-03
C1orf162	-1.18	5.49E-03
LOXL4	-1.29	5.70E-03
TLR3	-2.1	5.93E-03
CD48	-1.27	5.94E-03
KCNJ11	2.24	6.14E-03
BGN	1.17	6.29E-03
IL21R	-1.1	6.48E-03
TRIM9	1.01	6.58E-03
CXCL11	-2.35	6.60E-03
TNFRSF9	-1.47	6.66E-03
NRG1	1.75	6.68E-03
RIBC2	-1.56	6.77E-03
MAGI3	-1.31	6.85E-03
KIF25-AS1	-1.13	6.86E-03
DPY19L2P2	-2.62	6.95E-03
SIM2	1.47	7.02E-03
CCND2	1.5	7.13E-03
CASK	1.61	7.15E-03
ADAMTS1	1.07	7.15E-03
CHST13	-1.3	7.23E-03
NAV2-AS2	3.3	7.31E-03
GRIA3	-1.91	7.42E-03
KBTBD7	-1.03	7.42E-03
C7orf31	-1.23	7.45E-03
OCSTAMP	3.01	7.59E-03
SLC16A10	-1.23	7.62E-03
HES4	1.47	7.63E-03
SLC35E4	1.08	7.67E-03
ZNF37BP	-1.66	7.71E-03
LANCL3	-1.44	7.72E-03
CFH	1.26	7.81E-03
UPK3B	-2.91	7.98E-03
ETV7	-1.1	8.05E-03
LOC101929586	2.14	8.06E-03
IL10RB-AS1	-1.21	8.12E-03
GABBR2	1.49	8.21E-03

CYP26B1	2.64	8.31E-03
TMEM14E	-1.12	8.33E-03
GBP2	-1.05	8.33E-03
MTCL1	1.05	8.46E-03
BCL11B	1.09	8.76E-03
DBIL5P2	-3.5	8.79E-03
FCGR1C	-1.64	8.81E-03
RRS1-AS1	-2.29	8.86E-03
PMFBP1	-1.19	8.90E-03
C5AR2	1.04	9.08E-03
BRIP1	-1.15	9.20E-03
MIR612	-2.11	9.29E-03
NIM1K	-1.14	9.35E-03
SIGLEC11	-1.5	9.36E-03
MFAP5	1.35	9.42E-03
MTSS1	1.22	9.67E-03
PAMR1	1.8	9.72E-03
C1QTNF1	1.45	9.79E-03
ACKR3	1.39	1.00E-02
CLDN14	1.87	1.01E-02
LINC00968	-2.47	1.01E-02
BCAS4	2.44	1.01E-02
EVA1A	1.49	1.02E-02
DLGAP5	-1.35	1.03E-02
PTPN22	-1.47	1.03E-02
VAMP5	-1.34	1.04E-02
ZNF503	-1.13	1.04E-02
RASD2	-2.28	1.06E-02
TTC26	-1.03	1.08E-02
PLK2	1.03	1.08E-02
PRICKLE1	-1.09	1.09E-02
COL1A2	1.4	1.10E-02
THEM6	-1.28	1.13E-02
PLCD3	1.08	1.14E-02
TET1	-1.97	1.15E-02
GPR18	-1.05	1.16E-02
PROX2	-1.45	1.16E-02
ZNF700	-1.05	1.16E-02
FAM20A	-1.52	1.18E-02
PPP1R14C	1.14	1.19E-02
C19orf57	-1.57	1.19E-02
CDH11	1.45	1.20E-02
SYN1	1.32	1.20E-02

FAM161A	-1.97	1.20E-02
FAM65B	1.49	1.21E-02
EGR3	2.41	1.21E-02
GAPT	-2.21	1.22E-02
LINC00278	-1.35	1.27E-02
INHBA	1.38	1.27E-02
RGAG4	-1.03	1.28E-02
LOC101927543	-1.09	1.28E-02
DKFZP434K028	3.22	1.31E-02
CHN2	-1.74	1.31E-02
LOC101928790	-2.41	1.32E-02
ZFP14	-1.29	1.32E-02
USP32P2	-1.11	1.32E-02
COL5A1	1.34	1.32E-02
LOXHD1	1.02	1.32E-02
CD180	-1.49	1.34E-02
FBN1	1.39	1.37E-02
LOC642757	-2.31	1.37E-02
ZNF546	-1.02	1.38E-02
MROH9	1.66	1.39E-02
LDHAL6B	1.12	1.41E-02
MMP10	1.18	1.43E-02
CNN3	1.1	1.46E-02
LOC100288798	-2.41	1.47E-02
AMIGO2	1.28	1.47E-02
HESX1	-2.86	1.52E-02
LOC729683	1.09	1.54E-02
GBP1P1	-2.31	1.54E-02
LOC202181	-1.14	1.58E-02
HSPA4L	-1.09	1.61E-02
ZNF630	-3.42	1.65E-02
CDCA7	-1.05	1.67E-02
PAPPA	1.36	1.68E-02
CDH13	1.17	1.72E-02
HMMR-AS1	-3.09	1.73E-02
DNAH17-AS1	-1.2	1.76E-02
CTGF	1.4	1.80E-02
LOC641367	-1.83	1.81E-02
PTGER3	1.46	1.81E-02
MGC39584	-2.72	1.84E-02
PSMD6-AS2	-1.01	1.86E-02
FSTL1	1.13	1.87E-02
NKD1	-1.05	1.93E-02

KCNIP3	-2.08	1.94E-02
EGR2	2.09	1.99E-02
PLAC8	-1.18	1.99E-02
GCNT2	-1.33	2.08E-02
IL21R-AS1	-1.45	2.09E-02
MIR6835	2.1	2.09E-02
TRNP1	2.04	2.09E-02
SLC24A4	-1.38	2.10E-02
CCR7	1.31	2.11E-02
WNT7B	1.73	2.12E-02
MAP2	-1.32	2.17E-02
OCLM	-2.29	2.18E-02
SH3RF3	1.11	2.18E-02
SLC24A3	1.79	2.18E-02
THBS1	1.07	2.20E-02
ZNF44	-1.25	2.22E-02
MAP1LC3C	-2.46	2.25E-02
MYBL2	-1.03	2.27E-02
SPARC	1.3	2.31E-02
ZNF680	-1.04	2.37E-02
CENPM	-1.06	2.38E-02
RBM26-AS1	1.07	2.40E-02
LINC00964	1.5	2.43E-02
LOC148696	-1.03	2.45E-02
FGL2	-1.08	2.45E-02
CALB1	-2.65	2.46E-02
GVINP1	-2.08	2.47E-02
SHISA7	-2.22	2.49E-02
COL4A1	1.25	2.52E-02
SEZ6	-1.06	2.53E-02
MINOS1P1	-1.38	2.57E-02
ZNF519	-1.07	2.61E-02
COL3A1	1.29	2.62E-02
RAB20	-1.85	2.63E-02
ZNF331	-1.17	2.70E-02
COL1A1	1.18	2.71E-02
XIST	1.45	2.72E-02
CIITA	-2.63	2.73E-02
NSUN6	-1.19	2.73E-02
NKX3-1	1.9	2.73E-02
PCDHAC2	-1.1	2.77E-02
OLFML3	-1.37	2.79E-02
ZNF99	-3.13	2.79E-02

GPR141	-1.96	2.82E-02
XK	-1.86	2.87E-02
PLD4	-1.14	2.97E-02
GALNT9	1.23	2.97E-02
P2RX6	-1.81	2.98E-02
CSMD3	-2.82	3.01E-02
SERPINB2	1.91	3.05E-02
PTHLH	1.38	3.09E-02
FARP1	1.45	3.17E-02
KLF5	1.54	3.20E-02
LOC101927100	-1.19	3.20E-02
MEDAG	1.43	3.21E-02
FLRT1	-1.38	3.26E-02
LINC00342	-1.41	3.28E-02
DAPK2	-1.04	3.30E-02
CLDN11	1.11	3.31E-02
HAS2	1.17	3.33E-02
APLN	-1.61	3.36E-02
FTX	-1.29	3.41E-02
LOC100505736	-1.41	3.49E-02
ARHGAP29	1.05	3.51E-02
NHSL2	-1.67	3.55E-02
ABCG2	-1.12	3.55E-02
SCN5A	2.15	3.56E-02
PCOLCE	1.1	3.56E-02
NFIB	1.03	3.59E-02
TUSC3	1.49	3.68E-02
C16orf74	1.77	3.70E-02
LOC728637	-1.66	3.76E-02
BANK1	-2.06	3.79E-02
MIR7844	1.68	3.83E-02
TEDDM1	-1.79	3.88E-02
LOC158960	-1.79	3.90E-02
ERCC6L	-1.06	3.92E-02
C9orf139	-1.56	3.92E-02
SRSF12	-2.06	3.97E-02
NTN4	1.39	3.98E-02
ITGBL1	2.32	4.01E-02
CCDC171	-1.64	4.02E-02
COL4A2-AS1	1.5	4.02E-02
MCOLN3	-1.76	4.02E-02
ANKRD23	-1.11	4.03E-02
TGFBR3	1.04	4.05E-02

C22orf34	-1.19	4.10E-02
LOC101929723	-2.12	4.11E-02
ARHGAP31-AS1	-2.51	4.13E-02
ARSJ	1.45	4.13E-02
TMOD2	-1.19	4.16E-02
PRICKLE2	1.63	4.16E-02
HSPA6	-1.75	4.24E-02
TGFB3	1.54	4.27E-02
LOC101928766	2.08	4.27E-02
NR4A1	-1.59	4.30E-02
KLLN	-1.48	4.30E-02
RAB3B	1.14	4.32E-02
EPB41L4B	1.52	4.34E-02
ZNF485	-1.52	4.35E-02
FRMD5	-1.13	4.37E-02
CCL20	1.25	4.41E-02
LINC00702	1.71	4.44E-02
FPR3	-1.88	4.45E-02
PDE10A	2.38	4.45E-02
NOXA1	-2.32	4.51E-02
MIR7848	-2.51	4.52E-02
KIAA1841	-1.24	4.65E-02
ALX4	-2.06	4.85E-02
SLC46A2	-1.81	4.88E-02
EDIL3	1.09	4.89E-02
MIR4725	1.58	4.92E-02
SERPINE3	-1.02	4.95E-02

Table S4. Genes differentially expressed in THP-1 macrophages treated with Gap*CHROMR* versus GapCTRL after LPS stimulation for 3h.

Gene	log2FoldChange	padj
SNX3	-2.32	3.16E-145
CXCL10	-2.24	4.14E-134
CXCL11	-2.18	4.69E-112
SLC11A2	-1.93	6.14E-109
APP	-1.78	6.41E-98
SECISBP2	-2.57	5.73E-85
CCL2	-1.94	1.55E-67
IDO1	-1.7	1.75E-67
CXCL9	-2.87	7.73E-67
IL6	-2.49	4.82E-66
FGFR1OP	-2.59	3.61E-64
CCL8	-1.94	1.27E-60
NDUFV1	-1.59	7.37E-58
LOC1019270	-2.91	5.24E-56
GPX1	-1.37	7.79E-55
ME3	-2.47	1.52E-48
SMARCC1	-1.41	8.93E-45
IFIT2	-1.48	1.29E-44
COMMD10	-2.36	1.97E-44
CCDC30	1.6	1.21E-42
TNFSF10	-1.54	4.60E-42
CBWD1	-1.63	2.33E-40
RSAD2	-1.34	1.08E-39
RPL34	-1.16	2.29E-39
THBD	1.3	3.04E-39
CBWD2	-1.89	3.66E-37
TTC7B	-1.57	3.48E-36
GBP5	-1.07	8.82E-36
BNIP3L	-1.76	2.72E-35
IRGQ	1.05	2.98E-35
SAMD4A	-1.1	4.91E-35
IFIT1	-1.07	1.89E-34
IRG1	-1.15	2.20E-34
DOCK4	-1.24	7.32E-34
PDE3B	-1.17	2.36E-33
PHIP	-1.15	4.33E-33
RASA1	-1.17	9.57E-31
HERC5	-1.22	1.00E-30
ISG20	-1.29	1.10E-28

EPB41L2	-1.12	1.33E-28
USP18	-1.04	1.87E-28
C11orf31	-1.17	9.22E-28
CHID1	1.04	1.26E-27
GBP1	-1.01	3.53E-27
TRIB3	1.5	4.13E-27
FAF1	-1.24	8.23E-27
SMOX	1.12	9.98E-27
KREMEN1	1.05	3.98E-26
DAPP1	-1.33	5.17E-26
KLHL21	1.19	5.17E-26
GPR68	1.08	3.16E-25
MTMR6	-1.08	1.63E-24
CD274	-1.27	3.46E-24
ITGB8	-1.05	7.26E-24
EXOC4	-1.04	6.31E-23
SMC2	-1.26	9.32E-23
TRIM22	-1.02	1.16E-22
TNC	-1.44	1.52E-22
RB1	-1.09	2.55E-22
HAPLN3	-2.1	2.62E-22
USP53	1.09	1.22E-21
MT2A	-1.27	3.08E-21
IQCB1	-1.33	3.20E-21
CCL3L1	-1.02	5.93E-21
CCL3L3	-1.02	6.16E-21
MMP19	1.27	2.00E-20
GFPT2	-1.11	3.45E-20
IRF8	-1.04	2.17E-19
HS2ST1	-1.02	2.17E-19
CDCP1	1.28	9.11E-19
MYBPH	-1.18	1.02E-18
UBE2E2	-1.14	1.16E-18
STC1	1.72	2.23E-18
FCAR	1.37	3.67E-18
IFI44L	-2.61	3.84E-18
ETV6	-1.04	4.38E-18
CBWD5	-1.36	4.48E-18
GPR176	-1.49	4.66E-18
CCDC14	-1.11	5.26E-18
FAM212B	1.58	7.00E-18
MMP3	2.99	8.54E-18
S100A9	1.02	1.20E-17

SEMA4C	1.18	1.29E-17
RAPH1	1.19	2.71E-17
IL7	-1.03	3.30E-17
PAX5	-1.38	1.13E-16
ARID5A	-1.23	1.36E-16
CD70	-1.17	1.93E-16
MLKL	-1.08	1.13E-15
GBP1P1	-1.14	3.76E-15
NBPF12	1.05	7.40E-15
ANK2	-1.05	2.62E-14
LIF	-1.04	3.42E-14
KDM3A	1.02	5.85E-14
CCL1	-1.5	9.52E-14
UGGT2	-1.05	1.52E-13
LOC101927[illegible]	-1.12	1.54E-13
CXCL5	2.6	1.79E-13
SEMA4D	-1.09	2.46E-13
PLA2G4A	-1.08	3.05E-13
MET	1.02	5.32E-13
NRG2	-1.07	1.03E-12
NUAK2	-1.09	4.49E-12
ARAP2	-1.36	4.97E-12
RNVU1-19	-2.01	1.45E-11
CLDN23	-1	2.99E-11
IL24	1.3	3.09E-11
PDE4A	1.12	4.07E-11
ATE1	-1.02	1.72E-10
CHSY3	-1.68	3.91E-10
SECTM1	-1.53	4.13E-10
CCL7	-1.27	4.18E-10
VEGFC	-1.02	8.15E-10
NBPF20	1.1	1.43E-09
GRIN2D	1.1	1.64E-09
CBWD3	-1.81	1.65E-09
PCDH9	1.01	4.11E-09
FUK	-1.11	4.56E-09
DRAXIN	1.86	5.13E-09
GCA	-1.1	6.70E-09
KIFAP3	-1.05	7.10E-09
CDC42EP2	1.03	1.22E-08
EHD2	1.1	1.73E-08
UNC5C	-1.09	1.89E-08
CH25H	-1.71	1.91E-08

MAP2	-1.73	2.48E-08
ACRC	1.19	4.19E-08
MMP1	1.98	4.34E-08
MMP10	2.79	4.35E-08
DPYD-AS1	-1.02	4.97E-08
GCNT2	-1.71	5.16E-08
KLF4	-1.16	7.03E-08
STX1A	1.08	9.60E-08
KIAA1671	-1.4	1.33E-07
TNFAIP8L1	1.19	1.33E-07
KDM6A	-1.2	1.34E-07
PTPRO	-1.17	2.17E-07
DCAF4L1	1.7	2.34E-07
SLC44A5	-1.37	2.41E-07
KIF3A	1.05	2.70E-07
LOC102724[illegible]	-3.68	2.71E-07
TTC29	-1.1	2.96E-07
MMP8	1.71	3.10E-07
GTF2IRD1P	1.11	3.12E-07
VCAM1	-1.7	3.21E-07
MGAT4C	1.3	4.04E-07
RNU11	-1.62	5.15E-07
NRN1	-1.34	6.74E-07
PPBP	1.87	7.64E-07
TNFSF8	-1.38	8.00E-07
EMR3	1.67	8.76E-07
HTRA3	-1.42	9.43E-07
PRR12	1.05	1.01E-06
CBWD6	-1.6	1.12E-06
CFH	-1.41	1.13E-06
ICAM4	-1	1.69E-06
ADRB2	-1.02	1.70E-06
C11orf96	-1.39	1.81E-06
PELI3	1.01	1.92E-06
HESX1	-1.36	2.17E-06
CHI3L2	-1.24	2.29E-06
LOC100505[illegible]	-1.35	2.29E-06
ARC	-1.1	2.77E-06
IFITM1	-1.44	2.99E-06
RNU12	-1.45	3.03E-06
SHISA3	1.19	3.19E-06
ANXA2R	-1.01	3.62E-06
LOC103611[illegible]	1.13	3.96E-06

EPHB2	-1.2	4.43E-06
SORCS1	-1.28	5.26E-06
DPP4	-1.28	6.56E-06
FGD2	-1.13	6.75E-06
THY1	-2.02	7.49E-06
ZFP28	1.13	7.72E-06
IFIT1B	-1.17	1.07E-05
ABCA5	-1.38	1.08E-05
SMTNL1	-1	1.09E-05
WTAPP1	1.93	1.11E-05
FAM26E	-1.08	1.14E-05
NEXN	-1.17	1.28E-05
KRBA1	1.13	1.33E-05
CCR7	-1.04	1.47E-05
FRMD3	-1.28	1.95E-05
SRPX	-1.53	1.95E-05
AREG	1.33	2.11E-05
CD1D	-1.14	2.13E-05
SERPING1	-1.31	2.33E-05
PMFBP1	-1.32	2.41E-05
UTS2	-1.09	2.74E-05
CCNG2	1.29	3.90E-05
ZNF558	1	4.66E-05
TMPRSS13	-1.42	5.83E-05
FAM131A	1.09	5.93E-05
LOC101929	1.03	6.30E-05
FGL2	-1.24	7.39E-05
ZNF786	1.14	7.50E-05
OTOF	-1.75	1.02E-04
LAMP3	-1.04	1.11E-04
THSD4	-1.15	1.23E-04
CACNA1I	-1.44	1.27E-04
MMP12	1.3	1.29E-04
LAMA5	1.07	1.34E-04
SULT1C2	-1.3	1.49E-04
SOWAHC	1.06	1.70E-04
SLC22A17	-1.24	1.71E-04
SLC35F1	-1.32	2.60E-04
ZNF568	1.03	2.60E-04
LOC100133	1.31	2.72E-04
MIR4657	1.08	3.04E-04
TUBA8	-1.05	3.13E-04
RNVU1-20	-2.21	3.50E-04

LOC1019289	-1.25	3.89E-04
CHRM3	-1.94	4.02E-04
BANK1	-1.42	4.21E-04
MMP13	-1.96	4.27E-04
BTC	-1.3	4.34E-04
MARCO	-1.01	4.41E-04
ABTB1	1.19	4.52E-04
MYPOP	1.17	4.58E-04
IFNB1	-2	4.68E-04
ATP2B2	-1.07	5.12E-04
TM4SF1	1.62	6.02E-04
FCGR2B	-1.55	6.48E-04
CALN1	-1.07	6.75E-04
CYP2J2	-1.61	6.75E-04
GALNT18	-1.07	6.99E-04
VGLL2	-1.29	7.35E-04
ZNF610	1.01	7.46E-04
LINC00346	-1.14	8.33E-04
DAB2IP	-1.23	9.35E-04
HSH2D	-1.03	9.62E-04
MNDA	-1.4	1.05E-03
ROS1	-1.02	1.05E-03
GIMAP4	-1.37	1.07E-03
LARGE	-1.27	1.15E-03
CD101	-1.44	1.32E-03
PI15	-1.09	1.35E-03
EREG	1.27	1.36E-03
FAM46C	-1.54	1.56E-03
SPSB4	-1.5	1.60E-03
PRKXP1	1.08	1.97E-03
FXYD6-FX	-1.03	2.00E-03
SALL1	-1.06	2.08E-03
ACTN2	-1.33	2.27E-03
SEPT4	-1.15	2.36E-03
LILRB2	-1.03	2.42E-03
RASD1	-1.43	2.43E-03
RUFY4	-1.36	2.63E-03
PRTFDC1	-1.46	2.79E-03
AOC2	1.49	2.86E-03
RNF128	-1.37	3.31E-03
KIF5C	-1.16	3.40E-03
LILRA1	-1.3	3.40E-03
SCN3A	-1.86	3.42E-03

ACTA2	-2.31	3.53E-03
ADAMTS14	-1.21	3.76E-03
FCRLB	-1.16	3.91E-03
FAM47E	-1.48	4.13E-03
GMPR	-1.04	4.20E-03
MAMDC2	-1.62	4.34E-03
IL13RA2	1.5	4.44E-03
LPPR4	-1.86	4.44E-03
C11orf94	1.86	4.59E-03
FLJ39080	-1.38	4.64E-03
DYRK1B	1.12	4.78E-03
GRIK2	-1.29	4.85E-03
KCNH1	-1.74	5.53E-03
SCN5A	-1.32	5.55E-03
OVGP1	1.13	5.56E-03
IFI27	-1.85	6.01E-03
LYG1	1.87	6.30E-03
MAP7D2	1.48	6.33E-03
IL27	-1.33	6.36E-03
MIR6753	1.04	6.70E-03
LINGO1	-1.41	6.99E-03
PRUNE2	-1.28	7.31E-03
CACNA1B	-1.24	7.43E-03
SPARCL1	-1.06	7.54E-03
SUGCT	-1.8	7.62E-03
IFNLR1	-2	7.84E-03
GLI3	-1.53	7.88E-03
GRIK3	-1.55	8.42E-03
BATF3	-1.12	8.69E-03
CRIPAK	1.5	8.85E-03
DLG2	-1.58	9.09E-03
FFAR2	-1.2	9.19E-03
RNVU1-8	-1.6	9.83E-03
ZNF77	1.07	1.02E-02
SYT15	-1.01	1.06E-02
TTBK1	-1.21	1.11E-02
KCNIP2	-1.02	1.17E-02
FAXC	-1.78	1.23E-02
CAMK2B	-1.04	1.24E-02
DKFZP434I	1.1	1.24E-02
SELL	-2.01	1.30E-02
LRP1B	-1.44	1.32E-02
ZBP1	-1.78	1.38E-02

LOC101929099	-1.82	1.41E-02
LOC101927865	-1.09	1.56E-02
SNORD5	1.13	1.59E-02
BACE2	-1.51	1.60E-02
FHOD3	-1.11	1.63E-02
BBOX1	-1.32	1.65E-02
HS6ST3	-1.19	1.66E-02
ZNF10	1	1.74E-02
SERPIND1	1.49	1.80E-02
ASTN1	-1.47	1.80E-02
CFB	-1.56	1.83E-02
KCNA10	-1.57	1.88E-02
MBNL2	-1.15	1.95E-02
TMEM136	-1.03	1.95E-02
LOC100129:	1.08	1.96E-02
SLC26A3	1.33	1.96E-02
ABCG4	-1.46	1.97E-02
PADI2	-1.53	1.99E-02
SLC6A17	-1.46	2.02E-02
NPIPB3	1.15	2.07E-02
C16orf74	-1.52	2.14E-02
LEPREL2	-1.24	2.21E-02
SDK2	-1.05	2.22E-02
KCNIP1	-1.42	2.24E-02
ITK	-1.03	2.28E-02
CSMD2	-1.1	2.29E-02
MOXD1	-1.15	2.30E-02
FAT3	-1.41	2.31E-02
FAM227A	1.02	2.33E-02
SND1-IT1	1.04	2.38E-02
GPR31	-1.69	2.50E-02
CD300E	-1.15	2.51E-02
SEMA3F	-1.33	2.60E-02
ZNF300	-1.66	2.64E-02
SOCS2	-1.38	2.66E-02
IL20RB	1.13	2.66E-02
ADAMTSL1	-1.1	2.70E-02
CA8	-1.56	2.74E-02
LOC100506	-1.21	2.75E-02
SYT1	-1.17	2.82E-02
XDH	-1.3	2.89E-02
ELAVL4	-1.6	2.91E-02
CHDH	-1.94	2.94E-02

SYT17	-1.27	2.96E-02
AOC3	1.29	2.99E-02
TDRD9	1.07	3.09E-02
SEMA5A	-1.57	3.11E-02
SAMD12	-1.34	3.14E-02
LEPREL4	-1.01	3.33E-02
GCNT7	1.11	3.33E-02
CPXM1	-1.02	3.38E-02
NYNRIN	-1.5	3.41E-02
LINC00607	-1.3	3.47E-02
TLE2	-1.78	3.53E-02
TRIM29	-1.48	3.53E-02
MAGI2	-1.03	3.55E-02
C1QC	-1.48	3.57E-02
KLF2	-1.26	3.58E-02
PLSCR4	-1.18	3.72E-02
DRP2	-1.39	3.73E-02
CD34	-1.09	3.82E-02
HIST1H3E	-1.58	3.87E-02
SIDT1	-1.1	3.89E-02
LILRA5	-1.21	3.90E-02
LOC152225	1.11	4.08E-02
MAB21L3	1.08	4.16E-02
BCL2L14	-1.61	4.17E-02
NGF	-1.66	4.18E-02
CADM3	-1.54	4.20E-02
SLC24A2	-1.46	4.26E-02
PRKAG3	-1.49	4.28E-02
CYP2D7P	1.27	4.30E-02
NPM2	-1.12	4.31E-02
CDHR1	-1.39	4.37E-02
TRPC3	-1.58	4.38E-02
KIAA2022	-1.09	4.40E-02
SRGAP2-AS	-1.16	4.57E-02
FCRL5	-1.57	4.59E-02
MCF2L2	-1.3	4.62E-02
ZBED6CL	-1.13	4.65E-02
HIST2H2BA	-1.06	4.87E-02
LINC01366	-1.62	4.94E-02
ZNF709	1	4.95E-02
CMKLR1	-1.45	5.00E-02

Table S5. Genes differentially expressed in THP-1 macrophages overexpressing *CHROMR* versus an empty vector control by RT2 gene expression profiler

Gene	Fold change (log2)	P-value (log10)
IFI27	5.54	1.7010
IFITM1	3.98	1.1828
MX1	2.89	1.5409
MX2	2.51	1.5187
CXCL10	2.36	1.9146
IFIT1	2.18	1.0630
OAS2	2.14	1.1278
ISG15	2.11	1.4031
MNDA	2.10	1.5856
TNFSF10	2.09	2.9614
IFI6	1.98	1.0040
OAS1	1.94	1.1123
IFITM3	1.73	1.0246
IRF7	1.63	0.8418
DDX58	1.62	1.3135
STAT1	1.54	1.0587
BST2	1.44	1.3741
TLR9	1.31	0.5360
EIF2AK2	1.26	1.0214
MYD88	1.24	1.0224
IFI16	1.20	1.1422
GBP1	1.20	1.0900
IFIH1	1.04	0.6882
IRF9	0.96	0.8561
TIMP1	0.93	1.4447
IFIT3	0.89	0.6743
STAT2	0.84	0.6799
ADAR	0.70	0.5351
TAP1	0.62	0.9380
TLR3	0.59	0.4873
PML	0.54	0.4987
IFITM2	0.52	0.4813
PSME2	0.41	0.4247
TICAM1	0.40	0.6056
TYK2	0.39	0.4303
HLA-A	0.39	0.3388
MAL	0.32	0.4536
IFI30	0.28	0.2763
MET	0.25	0.3608
ISG20	0.25	0.2777
VEGFA	0.20	0.2554

IFNW1	0.20	0.1928
IRF3	0.19	0.2269
TRAF3	0.19	0.2063
IFNA1	0.18	0.1954
CD80	0.18	0.1479
CCL2	0.18	0.1029
IFNA2	0.16	0.1802
BAG3	0.16	0.0551
HLA-E	0.14	0.1458
TMEM173	0.10	0.0462
JAK2	0.06	0.0332
HLA-G	0.03	0.0039
NMI	0.00	0.0933
STAT3	0.00	0.0723
SHB	-0.03	0.0465
CD86	-0.04	0.1387
CASP1	-0.06	0.1303
HLA-B	-0.06	0.0233
CRP	-0.09	0.0923
IFNA4	-0.09	0.0923
IFNE	-0.09	0.0923
TLR8	-0.09	0.0608
IRF5	-0.12	0.0439
IL15	-0.12	0.0232
CCL5	-0.14	0.1585
CD70	-0.15	0.2267
IFNAR2	-0.15	0.0289
SOCS1	-0.15	0.0213
IRF2	-0.15	0.0045
IRF1	-0.20	0.0399
JAK1	-0.25	0.1785
CIITA	-0.27	0.1647
IL6	-0.27	0.0153
CAV1	-0.29	0.2659
IFNAR1	-0.32	0.0774
CDKN1B	-0.34	0.3993
SH2D1A	-0.45	0.2341
TLR7	-0.54	0.3492
PRKCZ	-0.54	0.1866
IFIT2	-0.84	0.4784
IFNB1	-1.15	0.5381
NOS2	-1.43	1.2135
IL10	-3.06	0.9766

Table S6. Table of oligonucleotides used in this study

qPCR

Primer	Sequence
hGAPDH_F	GAAGGTGAAGGTCGGAGTC
hGAPDH_R	GAAGATGGTGATGGGATTTC
hACTB_F	TGCGTTACACCCTTTCTTGA
hACTB_R	AAAGCCATGCCAATCTCATC
hHPRT1_F	ACCCCACGAAGTGTTGGATA
hHPRT1_R	AAGCAGATGGCCACAGAACT
CHROMR-univ_F	GCAGGAGCTTGAATTTCAGT
CHROMR-univ_R	TGTACTGAGTGGGCATTTAT
CHROMR-1_F	GATAAAAGGCCATCATCAGACA
CHROMR-1_R	CAAGCACAGGATTCCAAGGT
CHROMR-3_F	CTGGAGTGCAGTGCTGTGAT
CHROMR-3_R	ATCCCTTGAGGCCAAAAGTT
CHROMR-4_F	TGCCTTCTGTCTTTTGTCCA
CHROMR-4_R	GATTTGGGGCAAGTTGCTTA
CHROMR-5_F	GCGTTAGCCAGAATGGTTTC
CHROMR-5_R	GGACCACGTGAGGAAAAGTC
CHROMR-6_F	ATGCTCATGCCTCAGTCTCC
CHROMR-6_R	TGGAAAGCCATCTAAAAACCA
CHROMR-7_F	ATACCGGAGCCATGACAGAC
CHROMR-7_R	CGTCATCCTTGAATGTGTCATTA
HOTAIR_F	GGTAGAAAAAGCAACCACGAAGC
HOTAIR_R	ACATAAACCTCTGTCTGTGAGTGCC
NEAT1_F	TCGGGTATGCTGTTGTGAAA

Supplementary Index References

1. S. Heinz *et al.*, Transcription Elongation Can Affect Genome 3D Structure. *Cell* **174**, 1522-1536 e1522 (2018).
2. E. J. Hennessy *et al.*, The long noncoding RNA CHROME regulates cholesterol homeostasis in primate. *Nat Metab* **1**, 98-110 (2019).
3. S. Andrews, FastQC A Quality Control tool for High Throughput Sequence Data. (2008).
4. S. Chen, Y. Zhou, Y. Chen, J. Gu, fastp: an ultra-fast all-in-one FASTQ preprocessor. *Bioinformatics* **34**, i884-i890 (2018).
5. B. Langmead, S. L. Salzberg, Fast gapped-read alignment with Bowtie 2. *Nat Methods* **9**, 357-359 (2012).
6. H. Li *et al.*, The Sequence Alignment/Map format and SAMtools. *Bioinformatics* **25**, 2078-2079 (2009).
7. Y. Zhang *et al.*, Model-based analysis of ChIP-Seq (MACS). *Genome Biol* **9**, R137 (2008).
8. T. S. Carroll, Z. Liang, R. Salama, R. Stark, I. de Santiago, Impact of artifact removal on ChIP quality metrics in ChIP-seq and ChIP-exo data. *Front Genet* **5**, 75 (2014).
9. G. Yu, L. G. Wang, Q. Y. He, ChIPseeker: an R/Bioconductor package for ChIP peak annotation, comparison and visualization. *Bioinformatics* **31**, 2382-2383 (2015).
10. R. B. Stark, G. D. (2011) DiffBind: Differential Binding Analysis of ChIP-Seq Peak Data. Bioconductor.
11. C. Y. McLean *et al.*, GREAT improves functional interpretation of cis-regulatory regions. *Nat Biotechnol* **28**, 495-501 (2010).
12. S. Heinz *et al.*, Simple combinations of lineage-determining transcription factors prime cis-regulatory elements required for macrophage and B cell identities. *Mol Cell* **38**, 576-589 (2010).
13. C. Chu, J. Quinn, H. Y. Chang, Chromatin isolation by RNA purification (ChIRP). *J Vis Exp* 10.3791/3912 (2012).
14. A. M. Bolger, M. Lohse, B. Usadel, Trimmomatic: a flexible trimmer for Illumina sequence data. *Bioinformatics* **30**, 2114-2120 (2014).
15. H. Li, R. Durbin, Fast and accurate short read alignment with Burrows-Wheeler transform. *Bioinformatics* **25**, 1754-1760 (2009).
16. J. K. Eng, A. L. McCormack, J. R. Yates, An approach to correlate tandem mass spectral data of peptides with amino acid sequences in a protein database. *J Am Soc Mass Spectrom* **5**, 976-989 (1994).
17. M. V. Kuleshov *et al.*, Enrichr: a comprehensive gene set enrichment analysis web server 2016 update. *Nucleic Acids Res* **44**, W90-97 (2016).
18. A. Lachmann *et al.*, ChEA: transcription factor regulation inferred from integrating genome-wide ChIP-X experiments. *Bioinformatics* **26**, 2438-2444 (2010).
19. A. Armaos, A. Colantoni, G. Proietti, J. Rupert, G. G. Tartaglia, catRAPID omics v2.0: going deeper and wider in the prediction of protein-RNA interactions. *Nucleic Acids Res* **49**, W72-W79 (2021).

20. F. Agostini, D. Cirillo, B. Bolognesi, G. G. Tartaglia, X-inactivation: quantitative predictions of protein interactions in the Xist network. *Nucleic Acids Res* **41**, e31 (2013).
21. O. Kikin, L. D'Antonio, P. S. Bagga, QGRS Mapper: a web-based server for predicting G-quadruplexes in nucleotide sequences. *Nucleic Acids Res* **34**, W676-682 (2006).
22. A. R. Gruber, R. Lorenz, S. H. Bernhart, R. Neubock, I. L. Hofacker, The Vienna RNA websuite. *Nucleic Acids Res* **36**, W70-74 (2008).
